# Depletion of the transcriptional coactivators megakaryoblastic leukaemia 1 and 2 abolishes hepatocellular carcinoma xenograft growth by inducing oncogene-induced senescence

**DOI:** 10.1002/emmm.201202406

**Published:** 2013-07-29

**Authors:** Veronika Hampl, Claudia Martin, Achim Aigner, Sabrina Hoebel, Stephan Singer, Natalie Frank, Antonio Sarikas, Oliver Ebert, Ron Prywes, Thomas Gudermann, Susanne Muehlich

**Affiliations:** 1 Walther Straub Institute of Pharmacology and Toxicology, Ludwig-Maximilians-UniversityMunich, Germany; 2 Rudolf Boehm Institute of Pharmacology and Toxicology, Clinical Pharmacology, University of LeipzigGermany; 3 Institute of Pathology, University Hospital HeidelbergGermany; 4 Institute of Pharmacology and Toxicology, Technical University of MunichGermany; 5 Department of Medicine, Klinikum rechts der Isar, Technical University of MunichMunich, Germany; 6 Department of Biological Sciences, Columbia UniversityNew York, USA

**Keywords:** DLC1, MKL1, MKL2, MRTF, senescence

## Abstract

Megakaryoblastic leukaemia 1 and 2 (MKL1/2) are coactivators of the transcription factor serum response factor (SRF). Here, we provide evidence that depletion of MKL1 and 2 abolishes hepatocellular carcinoma (HCC) xenograft growth. Loss of the tumour suppressor deleted in liver cancer 1 (DLC1) and the subsequent activation of RhoA were prerequisites for MKL1/2 knockdown-mediated growth arrest. We identified oncogene-induced senescence as the molecular mechanism underlying the anti-proliferative effect of MKL1/2 knockdown. MKL1/2 depletion resulted in Ras activation, elevated p16 expression and hypophosphorylation of the retinoblastoma (Rb) protein in DLC1-deficient HCC cells. Interestingly, reconstitution of HuH7 HCC cells with DLC1 also induced senescence. Evaluation of the therapeutic efficacy of MKL1/2 knockdown *in vivo* revealed that systemic treatment of nude mice bearing HuH7 tumour xenografts with MKL1/2 siRNAs complexed with polyethylenimine (PEI) completely abolished tumour growth. The regression of the xenografts was associated with senescence. Importantly, PEI-complexed MKL1 siRNA alone was sufficient for complete abrogation of HCC xenograft growth. Thus, MKL1/2 represent promising novel therapeutic targets for the treatment of HCCs characterized by DLC1 loss.

## INTRODUCTION

Megakaryoblastic leukaemia 1/2 proteins (MKL1/2) function as coactivators of the transcription factor serum response factor (SRF) that regulates a broad range of cellular processes such as organization of the cytoskeleton, cell migration, cell growth and differentiation (Pipes et al, 2006). SRF is located at the nexus of RhoA- and MAPK-signalling pathways. Depending on the kind of signalling cascade activated, MKL1/2 or Ets-type ternary complex factors (TCFs) associate with SRF (Cen et al, 2003; Hill et al, 1995; Shaw & Saxton, 2003; Shaw et al, 1989). SRF binds to a highly conserved sequence CC(AT)_6_GG called the CArG box in the promoters of various immediate-early genes (IEGs) and genes involved in muscle-specific and contractile functions, actin dynamics and cell motility (Johansen & Prywes, 1995; Olson & Nordheim, 2010; Takeda et al, 1992; Treisman, 1986). IEG encoded proteins involved in cell cycle progression are aided by TCF cofactors (Buchwalter et al, 2004; Shaw et al, 1989). The association of SRF with the coactivators MKL1 (MRTF-A, MAL, BSAC) and MKL2 (MRTF-B) links gene transcription to changes in actin dynamics. The amino-termini of MKL1 and 2 contain RPEL domains that form a stable complex with monomeric G-actin and sequester MKLs in the cytoplasm. Actin polymerization liberates MKL1 and 2, leading to translocation of MKL1/2 to the nucleus and activation of gene expression through SRF (Miralles et al, 2003). Nuclear export of MKL1 is facilitated by G-actin binding and phosphorylation of MKL1 (Muehlich et al, 2008; Vartiainen et al, 2007). Such nuclear-cytoplasmic shuttling of MKLs is well established in fibroblasts and muscle cells. In contrast, in hepatocellular- and mammary-carcinoma cells, constitutive nuclear localization of MKL1/2 and activation of several tumour-relevant SRF target genes are observed (Muehlich et al, 2012). This is reminiscent of the MKL1 fusion protein with the RBM15 gene (a.k.a. OTT), which is a translocation found in acute megakaryoblastic leukaemia. RBM15-MKL1 is constitutively nuclear and activates SRF and its target genes (Descot et al, 2008; Ma et al, 2001; Mercher et al, 2009). The importance of MKL1/2 in metastasis was highlighted by a recent study of Medjkane and colleagues who demonstrated a requirement of MKL1/2 for experimental metastasis (Medjkane et al, 2009). Beyond that, however, the functional role of MKL1/2 in tumours has not been studied.

Mice lacking MKL1 are viable and fertile, but postpartum females fail to nurse their offspring due to defects in mammary myoepithelial cells (Li et al, 2006; Sun et al, 2006). Global deletion of MKL2 results in embryonic lethality owing to cardiovascular defects (Oh et al, 2005). These knockout studies have shown that MKL1/2 are not entirely redundant and are required at different developmental stages.

We recently reported that MKL1 and 2 mediate the effects of loss of the tumour suppressor deleted in liver cancer 1 (DLC1). DLC1 is a RhoGAP protein whose loss potentiates RhoA activity and thereby triggers MKL1/2 activation (Muehlich et al, 2012; Xue et al, 2008; Yuan et al, 1998). DLC1 is deleted in 50% of liver, breast, lung and 70% of colon cancers, almost as frequently as p53 mutations in these cancers (Lahoz & Hall, 2008; Xue et al, 2008). Despite its significance, there are currently hardly any targeted therapeutic options for cancers caused by loss of DLC1. Since the MKL/SRF signalling axis is of critical importance to cancerous transformation upon DLC1 loss, MKL1/2 might represent a promising pharmacological target for the therapy of DLC1-deficient cancers. However, therapeutic modulation of MKL signalling has not been explored so far.

In this study, we report that therapeutic knockdown of MKL1/2 abolishes tumour growth of DLC1-deficient hepatocellular carcinoma (HCC) xenografts by inducing oncogene-induced senescence.

## RESULTS

### Depletion of MKL1/2 provokes proliferation arrest in hepatocellular carcinoma cells with DLC1 loss

**Figure 1:**
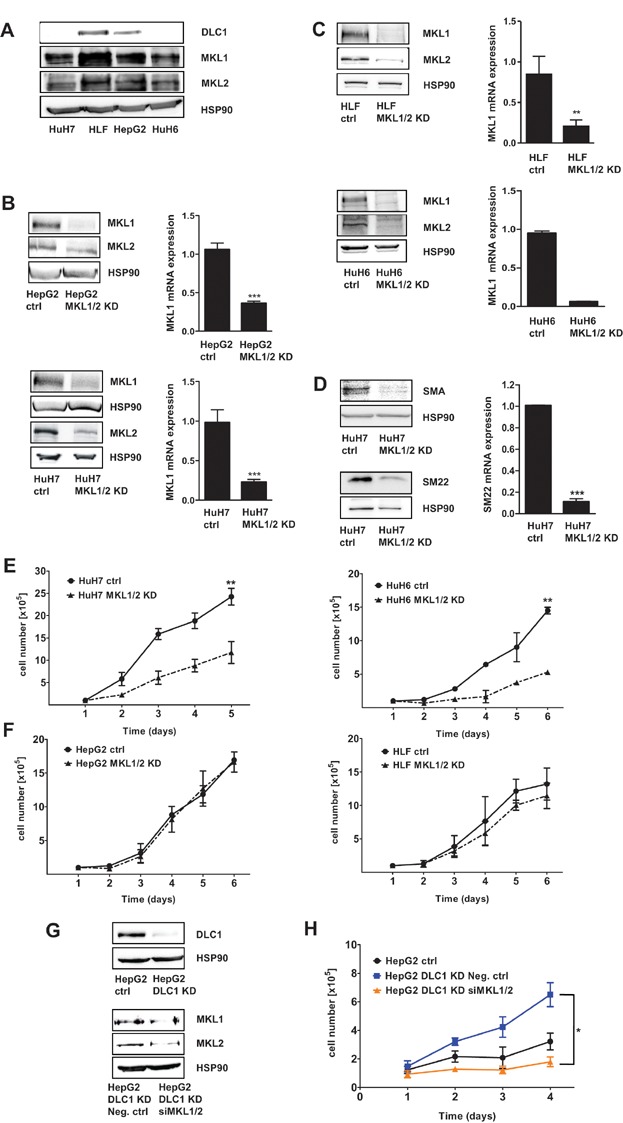
Depletion of MKL1/2 provokes proliferation arrest in hepatocellular carcinoma (HCC) cells with DLC1 loss **A.** Lysates of HuH7, HLF, HepG2 and HuH6 cells were immunoblotted with anti-DLC1, anti-MKL1, anti-MKL2 and anti-HSP90 antibody as a loading control.**B, C.** MKL knockdown efficiencies in the cell lines above were determined by immunoblotting using anti-MKL1, anti-MKL2 and anti-HSP90 antibodies or qRT-PCR. Values are mean ± SD (*n* = 3); ***p < 0.0001; ***p < 0.0001; **p = 0.0027.**D.** Lysates of HuH7 and HuH7 MKL1/2 Knockdown (KD) cells were immunoblotted with anti-SMA, anti-SM22 and anti-HSP90 antibodies. Relative SM22 mRNA expression was measured by qRT-PCR. Values are mean ± SD (*n* = 3); ***p < 0.0001.**E.** The DLC1-deficient HuH7 and HuH6 cells expressing control shRNA or MKL1/2 shRNA were counted daily for 6 days. Values are mean ± SEM (*n* = 3); **p = 0.0021; **p = 0.0016.**F.** Endogenous DLC1 expressing HepG2 and HLF cells were transduced with control shRNA or MKL1/2 shRNA and counted daily for 6 days. Values are mean ± SEM (*n* = 3).**G.** HepG2 cells stably expressing control shRNA or DLC1 shRNA were immunoblotted with anti-DLC1 and anti-HSP90 antibodies. HepG2 cells expressing DLC1 shRNA were transfected with either Neg. ctrl siRNA or siRNA MKL1/2 (50 nM). After 72 h, lysates were subjected to immunoblotting using anti-MKL1, anti-MKL2 and anti-HSP90 antibodies.**H.** The indicated cells were counted daily for 4 days. Values are mean ± SEM (*n* = 3); *p = 0.0356.

We recently reported that MKL1/2 knockdown in HuH7 hepatocellular cancer cells impairs cell proliferation (Muehlich et al, 2012). To further investigate whether loss of the tumour suppressor DLC1 is required for the proliferation arrest upon MKL1/2 knockdown, we depleted MKL1 and 2 in 4 different hepatoma cell lines (HuH7, HLF, HepG2 and HuH6). While all cell lines express MKL1/2, HepG2 and HLF differ from HuH6 and HuH7 cells in their expression of DLC1 (Fig 1A). An shRNA vector targeting MKL1 and 2 was introduced into these cells using lentiviral transduction to enable long-term analysis of cell proliferation. Control cells contained a lentiviral vector with an shRNA sequence that does not target known genes. The lentiviral shRNA vectors coexpressed a puromycin gene that allowed selection of transduced cells within 7 days. Using this protocol, an overall knockdown efficiency of 50–80% was achieved on MKL1/2 mRNA and protein levels (Fig 1B, C; Supporting Information Fig S1). To validate functional MKL1/2 depletion, we examined the expression of the well established MKL/SRF target genes smooth muscle actin (SMA) and transgelin (SM22). MKL1/2 depletion almost completely abolished SMA protein and SM22 mRNA expression (Fig 1D, Supporting Information Fig S2). We observed reduced cell proliferation in DLC1-deficient HuH7 and HuH6 cells expressing MKL1/2 shRNA as compared to control shRNA populations (Fig 1E). No significant changes in proliferation were detected in DLC1-expressing HepG2 and HLF cells transduced with MKL1/2 shRNA and control shRNA (Fig 1F).

To ultimately test whether DLC1 deficiency plays a causal role in the MKL1/2 knockdown-mediated growth arrest, an shRNA vector targeting DLC1 was introduced in HepG2 cells, resulting in a knockdown efficiency of 83% on DLC1 protein levels (Fig 1G). We observed enhanced cell proliferation in HepG2 cells expressing DLC1 shRNA as compared to control shRNA populations. Knockdown of MKL1/2 in HepG2 DLC1 shRNA expressing cells abolished the pro-proliferative effect of DLC1 knockdown (Fig 1H), demonstrating that DLC1 deficiency renders HepG2 cells responsive to the effect of MKL1/2 knockdown on cell proliferation.

These results indicate that depletion of MKL1 and 2 impairs cell proliferation of HCC cells lacking DLC1.

### Increased RhoA activity is required for reduced proliferation upon MKL1/2 knockdown

**Figure 2:**
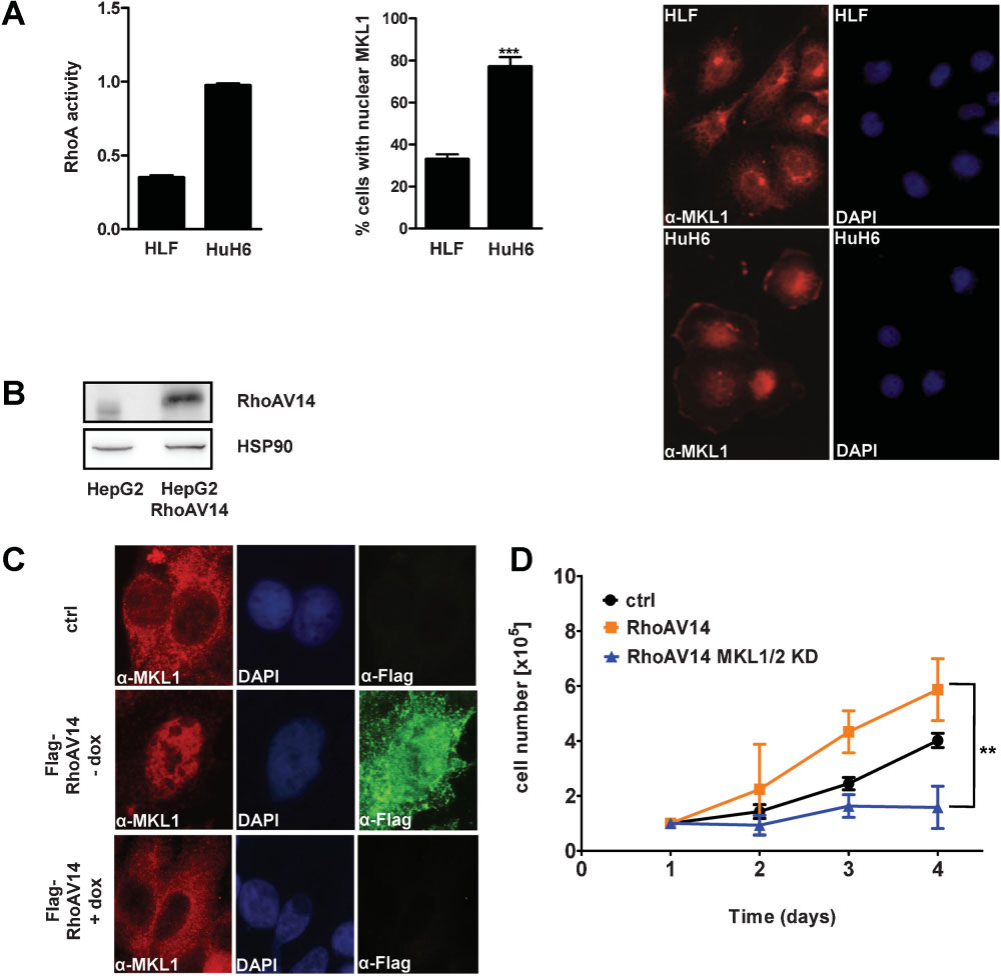
Increased RhoA activity is required for reduced proliferation upon MKL1/2 knockdown Active RhoA-GTP levels of HLF and HuH6 cells were assessed by an ELISA assay. HLF and HuH6 cells were stained with anti-MKL1 antibody for immunofluorescence analysis. Nuclei were counterstained with DAPI. Subcellular localization of endogenous MKL1 was scored as predominantly nuclear in 100 cells per experiment. Representative images are shown. Values are mean ± SD (*n* = 3); ***p = 0.001.HepG2 Tet-off cells expressing pRevTRE control or pRevTRE FLAG-RhoAV14 vector were lysed and immunoblotted with anti-RhoA and anti-HSP90 antibodies.HepG2 Tet-off and HepG2 Tet-off RhoAV14 cells with and without 0.5 µg/mL doxycycline (dox) were immunostained with anti-MKL1 and anti-FLAG antibodies. Nuclei were stained with DAPI.HepG2 Tet-off cells expressing pRevTRE RhoAV14 were transduced with either control shRNA or MKL1/2 shRNA and counted daily for 4 days. Values are mean ± SEM (*n* = 3); **p = 0.0051.

Since MKL1/2 knockdown exerted its inhibitory effect on cell proliferation in DLC1-deficient, but not in DLC1-expressing cells, we hypothesized that the enhanced RhoA activity caused by DLC1 loss is a prerequisite for the observed proliferation arrest. According to our previous study, loss of DLC1 in HuH7 cells leads to enhanced RhoA activation and MKL1/2 nuclear localization, whereas DLC1-expressing HepG2 cells display low RhoA activity and cytoplasmic MKL1 distribution (Muehlich et al, 2012). Accordingly, we observed increased RhoA activation and MKL1/2 nuclear accumulation in HuH6 cells (characterized by impaired proliferation after MKL1/2 depletion), but not in HLF cells (Fig 2A). Therefore, HLF and HepG2 cells have little active nuclear MKL1/2. These results are consistent with our hypothesis of an essential role of RhoA activity in MKL1/2-dependent growth.

To lend further credence to this model, constitutively active RhoA (RhoAV14) was introduced into HepG2 cells with low RhoA activity by a relatively weak Tet-off-regulated promoter (Fig 2B). As expected, expression of constitutively active RhoA by withdrawal of doxycyline led to an accumulation of MKL1 in the nucleus of HepG2 cells (Fig 2C). Addition of doxycycline caused MKL1 redistribution to the cytoplasm (Fig 2C, bottom). In contrast to our results in wildtype cells, the knockdown of MKL1/2 in RhoAV14-expressing HepG2 cells significantly impaired cell proliferation (Fig 2D; *cf*. Fig 1E).

These data strongly suggest that increased RhoA activity is indispensable for proliferation arrest caused by MKL1/2 knockdown.

### Loss of MKL1/2 induces senescence phenotypes

To characterize the nature of the decrease in proliferation induced by MKL1/2 depletion, we first assessed the effect of reduced MKL1/2 expression on apoptosis by measuring activated caspase3 with a specific antibody. The caspase3 family of cysteinyl proteases has been implicated as key mediators of apoptosis in mammalian cells (Nicholson et al, 1995). Active caspase3 was observed in HuH7 cells treated with the pro-apoptotic drug staurosporine as positive control, but not in HuH7 MKL1/2 KD cells, indicating that apoptosis did not account for the proliferation arrest (Supporting Information Fig S3).

**Figure 3:**
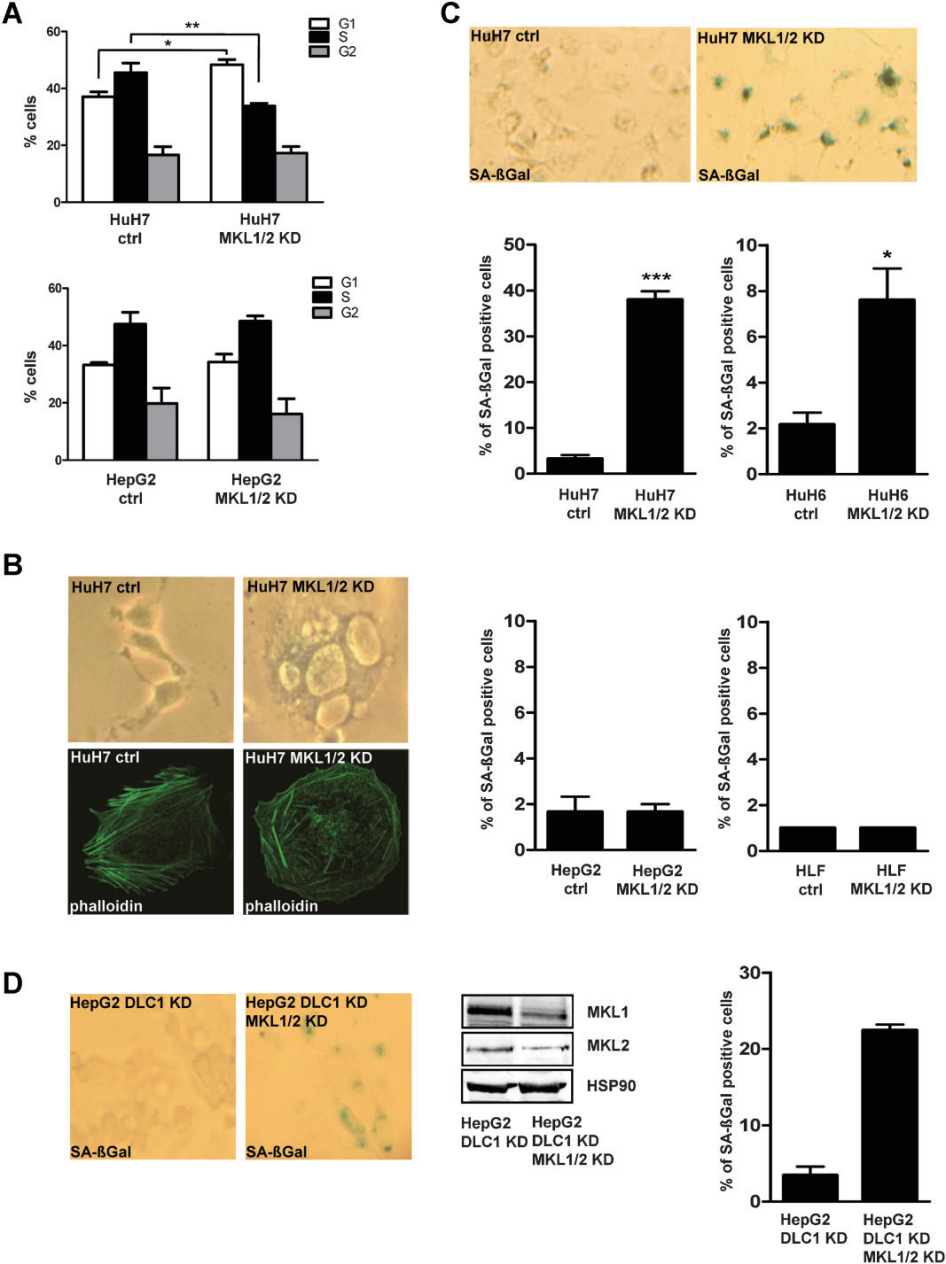
Loss of MKL1/2 induces senescence phenotypes Cell cycle phase distribution of HuH7 and HepG2 cells expressing control shRNA or MKL1/2 shRNA was examined by flow cytometry. Values are mean ± SD (*n* = 3); *p = 0.011; **p = 0.0042.The cell morphology and the actin cytoskeleton of HuH7 and HuH7 MKL1/2 KD cells were visualized after Alexa Fluor 488 phalloidin binding.At day 7 posttransduction senescence associated β-galactosidase staining was performed in the indicated cell lines. Values are mean ± SD (*n* = 3); ***p < 0.0001; *p = 0.02.HepG2 cells expressing DLC1 shRNA and MKL1/2 shRNA were immunoblotted with anti-MKL1, anti-MKL2 and anti-HSP90 antibodies. Senescence associated β-galactosidase staining was performed. Values are mean ± SD (*n* = 2).

Flow cytometry analysis revealed that HuH7 MKL1/2 KD cells had a larger G1 phase and, correspondingly, a smaller S phase population than control HuH7 cells. In contrast, MKL1/2 KD had no effect on the cell cycle of the DLC1-positive HepG2 cells (Fig 3A).

The increased cell population in the G1 phase and the flat, enlarged, vacuole-rich morphology devoid of actin stress fibres (Fig 3B) were reminiscent of senescent cells. Therefore, we set out to assess established senescence markers in HuH7 cells and monitored senescence-associated β-galactosidase activity. There was a significant increase in SA-β-gal positive HuH7 cells upon MKL1/2 knockdown (4% *vs*. 38%). A similar result was obtained in HuH6 MKL1/2 KD cells. In contrast, there was no increase in SA-β-gal positive cells upon MKL1/2 KD in HepG2 and HLF cells (Fig 3C). Positive SA-β-gal activity upon MKL1/2 KD was detectable only after depletion of DLC1 expression in HepG2 DLC1/MKL1/2 double knockdown cells (Fig 3D). Thus, cellular senescence due to MKL1/2 KD was commensurate with the observed suppression of cell proliferation caused by MKL1/2 KD in DLC1-negative cells and is a plausible explanation for the cellular phenotype.

### Loss of MKL1/2 leads to oncogene-induced senescence

**Figure 4:**
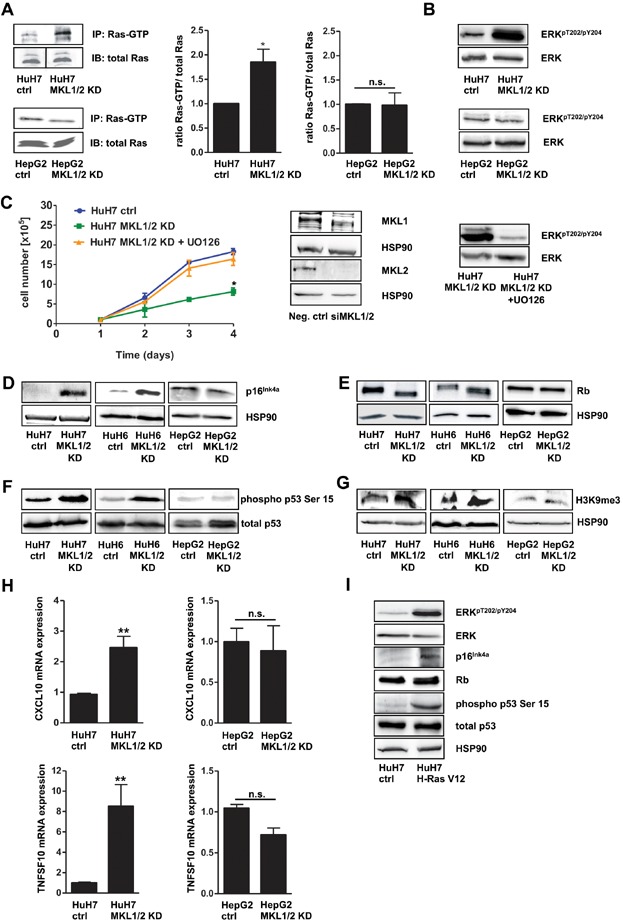
Loss of MKL1/2 leads to oncogene-induced senescence HuH7, HuH7 MKL1/2 KD, HepG2 and HepG2 MKL1/2 KD cells were immunoprecipitated with anti-active Ras antibody and immunoblotted with anti-Ras antibody. Equal amounts of lysates were directly immunoblotted with anti-Ras antibody. The black line delineates a boundary caused by juxtaposing lanes that were non-adjacent in the same gel. The relative ratio of active Ras versus total Ras was quantitated and graphically depicted on the right. Values are mean ± SD; *p = 0.046.Lysates of HuH7 and HepG2 cells with control shRNA or MKL1/2 shRNA were immunoblotted with anti-ERK^pT202/pY204^ and total anti-ERK antibodies.Neg. ctrl siRNA or MKL1/2 siRNA (50 nM) expressing HuH7 cells, treated with or without 10 µM U0126 were counted daily for 4 days. Values are mean ± SEM (*n* = 3); *p = 0.02. Lysates of the indicated cells were subjected to immunoblotting using anti-MKL1, anti-MKL2, anti-HSP90, anti-ERK^pT202/pY204^ and total anti-ERK antibodies.Lysates of the indicated cells were immunoblotted with anti-p16^Ink4a^ and anti-HSP90 antibodies.Lysates of the indicated cells were immunoblotted with anti-Rb and anti-HSP90 antibodies.Lysates of the indicated cells were immunoblotted with anti-phospho p53 serine 15 and total anti-p53 antibodies.Lysates of the indicated cells were immunoblotted with anti-H3K9me3 and anti-HSP90 antibodies.Relative CXCL10 and TNFSF10 mRNA expression of HuH7 and HepG2 cells was measured by qRT-PCR. Values are mean ± SD (*n* = 3); **p = 0.0034; **p = 0.0079.HuH7 cells were infected with pBabe H-Ras V12 or pBabe control vector. At day 5 posttransduction, cells were lysed and subjected to immunoblotting using anti-ERK^pT202/pY204^, total anti-ERK, anti-p16^Ink4a^, anti-phospho p53 serine 15, total anti-p53 and anti-HSP90 antibodies.

Two different categories of cellular senescence can be distinguished: replicative and premature senescence. The former refers to the limited number of replication cycles a cell can undergo due to end replication problems, whereas the latter occurs despite intact telomeres and is induced by acute cell stress inflicted by overactive oncogenes like Ras and Myc, DNA-damaging agents, hypoxia or by metabolic stress. Oncogenic stress can elicit senescence via at least three different routes: the Arf/p53/p21-, the p16/pRb- and the DNA damage response (DDR)-pathways (Larsson, 2011). Analysis of the different senescence pathways revealed that HuH7 MKL1/2 KD cells display an increase in active Ras protein, as determined by immunoprecipitation with an antibody that specifically recognizes GTP-bound Ras (Fig 4A). There was no effect on Ras activity in HepG2 cells lacking senescence induction by MKL1/2 KD (Fig 4A).

Since Ras activates the Raf-MEK1-ERK1/2 pathway, we also tested for activation of ERK1/2 by examining its phosphorylation status. ERK1/2 phosphorylation was strongly increased upon MKL1/2 knockdown in HuH7 and HuH6 cells, but not in HepG2 and HLF MKL1/2 KD cells with normal proliferative capacity (Fig 4B and Supporting Information Fig S4). Next we tested whether the activation of the Ras-Raf-MEK1-ERK1/2 pathway is responsible for MKL1/2-mediated proliferation arrest by administering the MEK1 inhibitor U0126 to prevent ERK1/2 activation (Fig 4C). The efficiency of U0126 treatment was determined by its ability to inhibit ERK1/2 phosphorylation (Fig 4C, right). We found that the anti-proliferative effect of MKL1/2 knockdown in HuH7 cells was abolished in the presence of U0126 (Fig 4C), in contrast to the general assumption of ERK1/2 activity serving a pro-proliferative role. The effect of U0126 further suggests a role for the Ras-ERK cascade in proliferation arrest due to MKL1/2 depletion.

Testing other senescence markers, we observed accumulation of p16^INK4a^ and hypophosphorylation of Retinoblastoma (Rb) protein in HuH7 MKL1/2 KD and HuH6 MKL1/2 KD cells, but not in HepG2 MKL1/2 KD cells (Fig 4D, E). Changes in the mobility of Rb during gel electrophoresis were used as an indicator for Rb phosphorylation (Connell-Crowley et al, 1997). Rb shifted to a faster migrating, hypophosphorylated form in HuH7 and HuH6 cells, whereas there was no change in mobility in HepG2 cells (Fig 4E). To examine whether the expression of p16^INK4a^ is essential for senescence induction by MKL1/2 depletion, we performed siRNA-mediated p16^INK4a^ knockdown prior to lentiviral transduction with MKL1/2 shRNA and analysed the phosphorylation status of Rb. Hypophosphorylation of Rb was observed upon MKL1/2 knockdown, but not upon p16^INK4a^/MKL1/2 double knockdown, suggesting that p16^INK4a^ expression is required for the MKL1/2 knockdown-mediated senescence response (Supporting Information Fig S5).

To further corroborate that depletion of MKL1/2 induces senescence, we evaluated additional senescence markers for DNA damage and senescence-associated heterochromatin foci (SAHF) (Di Micco et al, 2006; Narita et al, 2006). We observed p53 Ser15 phosphorylation as an indicator for DNA damage and an accumulation of Histone H3 methylated on lysine 9 in SAHF upon MKL1/2 depletion in HuH7 and HuH6 cells, confirming that these cells are in senescence (Fig 4F and G).

The induction of senescence requires several secreted factors, referred to as ‘senescence-messaging secretome (SMS)’ (Kuilman and Peeper, 2009). To identify SMS factors, we performed a microarray in HuH7 and HuH7 MKL1/2 KD cells and found a significant increase in chemokine (C-S-C motif) ligand 10 (CXCL10) and TNFSF10 mRNA expression in HuH7 and HuH6 MKL1/2 KD cells (Fig 4H and Supporting Information Fig S6). No change was observed in HepG2 cells depleted of MKL1/2.

Since Ras and its downstream targets are well known inducers of oncogene-induced senescence in primary cells, but not in cancer cells, we tested whether expression of active Ras is able to induce oncogene-induced senescence in HuH7 and HuH6 cells by retroviral transduction of an activated ras allele (H-*ras*V12). On the fourth day postinfection, enhanced ERK phosphorylation, p16 expression, hypophosphorylation of Rb and phosphorylation of p53 at Ser15 was detectable in HuH7 and HuH6 cells (Fig 4I and Supporting Information Fig S7), indicating that constitutive expression of active Ras induces senescence in HuH6 and HuH7 cells.

We conclude that HuH7 and HuH6 HCC cells possess characteristic hallmarks associated with oncogene-induced senescence following depletion of MKL1/2.

### Induction of cellular senescence in HuH7 cells after reconstitution of DLC1

**Figure 5:**
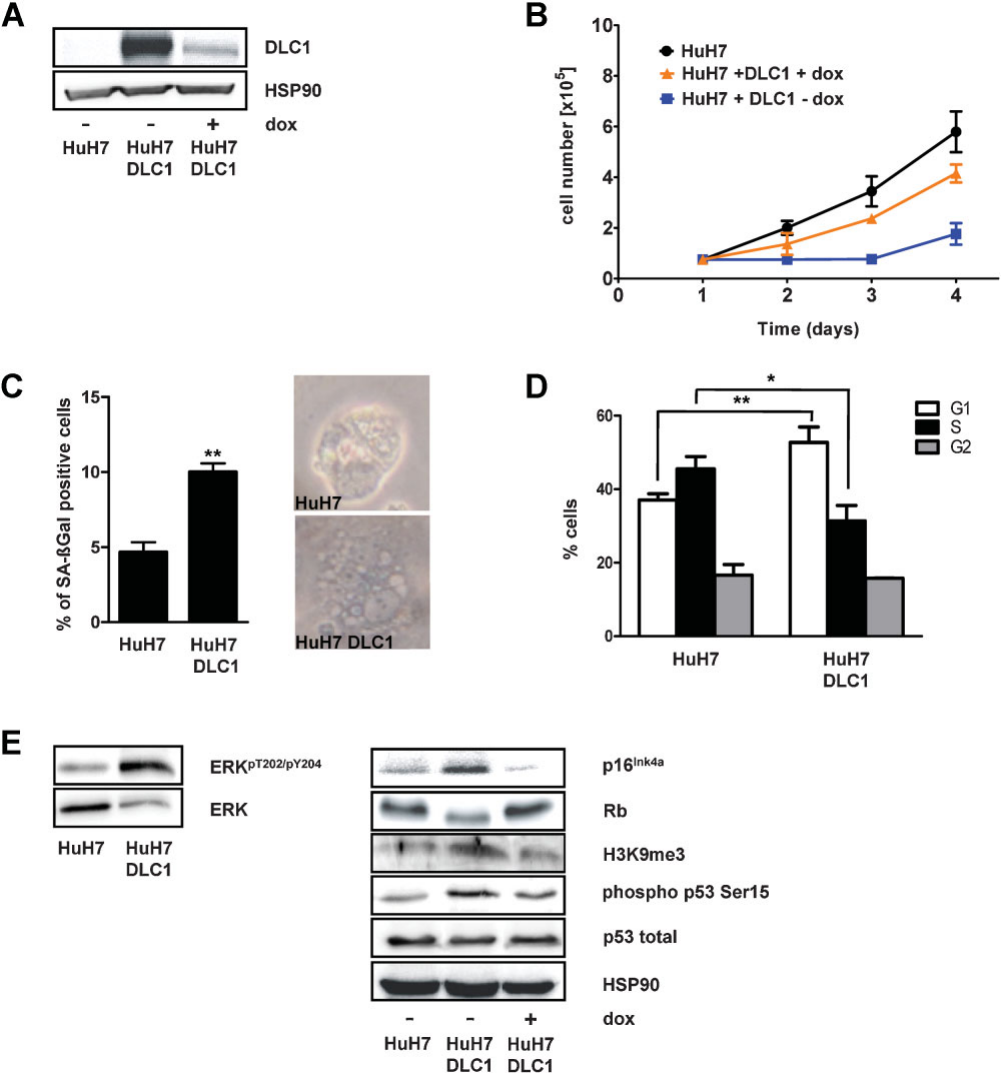
Induction of cellular senescence in HuH7 cells after reconstitution of DLC1 HuH7 Tet-off and HuH7 Tet-off DLC1 cells with and without 0.5 µg/mL doxycycline (dox) were subjected to immunoblotting with anti-DLC1 and anti-HSP90 antibodies.The indicated cell lines were counted daily for 4 days. Values are mean ± SEM (*n* = 2).Senescence associated β-galactosidase staining was performed with HuH7 Tet-off and HuH7 Tet-off DLC1 cells upon withdrawal of doxycyline, and a representative image of the cell morphology captured. Values are mean ± SD (*n* = 3); **p = 0.0038.Cell cycle phase distribution of HuH7 Tet-off and HuH7 Tet-off DLC1 cells in the absence of doxycycline was examined by flow cytometry. Values are mean ± SD (*n* = 3); **p = 0.009; *p = 0.02.Lysates of HuH7 Tet-off and HuH7 Tet-off DLC1 cells cultivated without and with doxycycline were immunoblotted with anti-ERK^pT202/pY204^, total anti-ERK, anti-p16^Ink4a^, anti-Rb, anti-H3K9me3, anti-phospho p53 serine 15, total anti-p53 and anti-HSP90 antibodies.

Since oncogene-induced senescence suppresses tumour growth, we asked whether in DLC1-negative cells MKL1/2 downregulation may substitute for the tumour-suppressive actions of DLC1. To address this question, we established a stable Tet-off HuH7 cell line expressing GFP-DLC1 in the absence of doxycycline (Fig 5A). We observed that HuH7 cells stopped to proliferate upon withdrawal of doxycycline and concomitant expression of DLC1 (Fig 5B). There was also a decrease in growth in these cells with doxycycline, possibly due to low level expression of DLC1. Interestingly, expression of DLC1 led to a significant increase in the percentage of SA-β-gal positive cells that exhibited the characteristic vacuole-rich morphology of senescent cells (Fig 5C). Flow cytometry analysis revealed a pronounced increase in G1 phase cells (Fig 5D). Reconstitution of DLC1 upon withdrawal of doxycycline also resulted in enhanced phosphorylation of ERK1/2, increased p16^INK4a^ expression, hypophosphorylation of Rb, methylation of histone H3 at lysine 9 and p53 phosphorylation on serine 15 (Fig 5E). Addition of doxycycline prevented the induction of the latter senescence markers (Fig 5E).

The results presented in Fig 5 are consistent with the concept that DLC1 exerts its tumour suppressive effects by inducing cellular senescence via the p16/pRb pathway. Together with our previous results, these data suggest that depletion of MKL1/2 mimics the effects of DLC1 reconstitution.

### Anti-tumour effects of therapeutic MKL1/2 knockdown *in vivo*

The results obtained so far indicate that MKL1/2 may be valuable targets for the therapy of DLC1-deficient cancers. To assess the therapeutic efficacy of MKL1/2 knockdown *in vivo*, we aimed at treating well-established tumours based on wildtype cells. To this end, we employed polyethylenimine (PEI) complexation of siRNAs as an efficient tool for *in vivo* siRNA delivery (Hobel & Aigner, 2010). We used an MKL1/2 siRNA targeting both MKL1 and 2, a combination of MKL1 and MKL2-specific siRNAs and MKL1 siRNA alone. The MKL1/2 sequence corresponded to the MKL1/2 shRNA sequence. Knockdown efficiencies of MKL siRNAs were determined *in vitro* by immunoblotting (Fig 4C, Supporting Information Fig S8). To validate functional depletion of MKL1 and 2, we also analyzed the expression of the well-known target genes transgelin (SM22) and SMA. SM22 mRNA expression was significantly reduced in response to MKL1 + 2 siRNA treatment. Likewise, SMA protein expression was strongly downregulated in HuH7 cells transfected with MKL1/2 siRNA or MKL1 siRNA (Supporting Information Fig S9).

**Figure 6:**
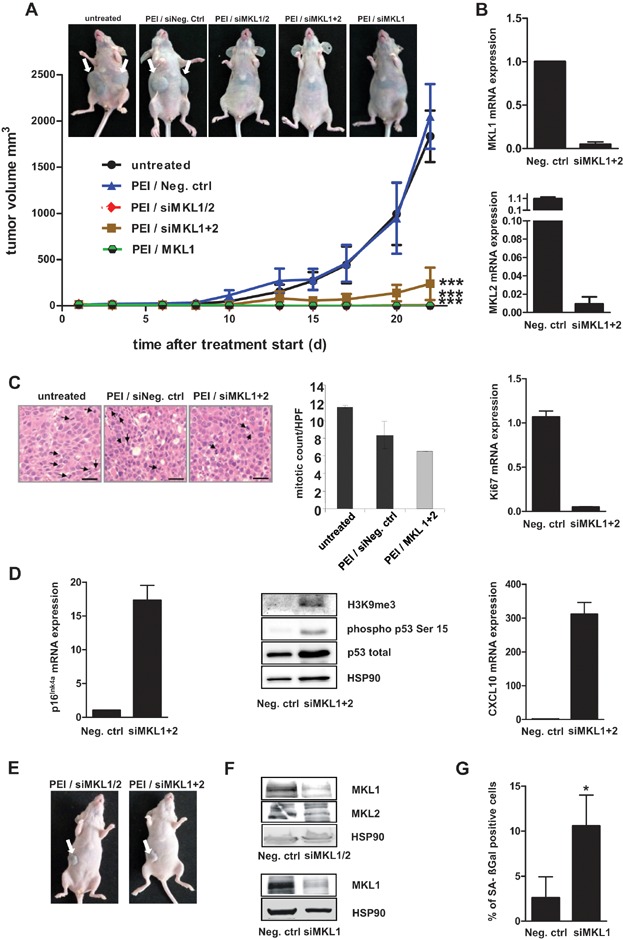
Anti-tumour effects of therapeutic MKL1/2 knockdown *in vivo.* Upon establishment of s.c. HuH7 tumour xenografts, tumour-bearing mice were randomized and treated three times a week by systemic injection of 15 µg PEI-complexed siRNAs. Values are mean ± SEM; ***p < 0.001 (see upper panel for representative examples of mice, tumours are indicated by arrows).MKL knockdown efficiencies were determined by qRT-PCR. Values are mean ± SD of two RNA isolations from one tumour xenograft.The averaged mitotic count/high power field (mitosis/HPF) of the indicated tumours was determined by histological evaluation (right panel). Arrows indicate mitotic figures in corresponding H&E stained tumour specimens (left panel). Scale bare represents 100 µm. Relative Ki67 mRNA expression of HCC xenografts treated with Neg. ctrl siRNA and MKL1 + 2 siRNA was measured by qRT-PCR. Values are mean ± SD.Relative p16^Ink4a^ mRNA expression of HCC xenografts treated with Neg. ctrl siRNA and MKL1 + 2 siRNA was determined by qRT-PCR. Values are mean ± SD of two RNA isolations from one xenograft specimen. Lysates of the indicated HCC xenografts were immunoblotted with anti-H3K9me3, anti-phospho p53 serine 15, total anti-p53 and anti-HSP90 antibodies. Relative CXCL10 mRNA expression was examined by qRT-PCR. Values are mean ± SD of two RNA isolations from one xenograft specimen.Images of recurrent tumours, indicated by arrows, in the PEI/MKL1/2- and PEI/MKL1 + 2-specific treatment group.MKL knockdown efficiencies in the lysates of the recurrent tumours were determined by immunoblotting using anti-MKL1, anti-MKL2 and anti-HSP90 antibodies.Recurrent HCC xenografts treated with MKL1 siRNA were excised, cultivated *in vitro* for 6 days and stained for senescence associated β-galactosidase activity. Values are mean ± SD (*n* = 3); *p = 0.029.

We generated subcutaneous tumour xenografts by injecting HuH7 cells into athymic nude mice. Upon formation of solid tumours, mice were treated systemically by intraperitoneal (i.p.) injection of PEI/siRNA complexes three times a week. No treatment at all or treatment with PEI-complexed control siRNA that does not target known genes served as negative control conditions. Strikingly, tumour growth was completely abolished in the MKL1/2- and MKL1-specific treatment groups. Comparably, in the MKL1 + 2-specific treatment group, only one out of six mice remained bearing a tumour (Fig 6A). In the xenografts treated with MKL1 + 2 siRNA, immunoblotting and immunohistochemistry upon termination of the experiment on day 28 after injection of HuH7 cells revealed strongly reduced MKL1 and 2 mRNA expression (Fig 6B) and a concomitant lower proliferation rate, as determined by Ki-67 mRNA expression and the mitotic count (Fig 6C). In order to confirm that the regression of the xenografts is associated with senescence in the tumours treated with MKL1 + 2 siRNA, we determined p16^INK4a^ expression. P16^INK4a^ mRNA expression was significantly elevated in tumours of mice treated with MKL1 + 2 siRNA. Furthermore, we were able to verify the other candidate senescence markers shown in Fig 4 *in vivo*, such as an accumulation of histone H3 methylated on lysine 3 (H3K9me3), phosphorylation of p53 at serine 15 and enhanced CXCL10 expression as a component of the senescence-associated secretome (Fig 6D). Tumour-free mice were monitored for another 4 weeks to test for tumour recurrence. The MKL1-specific treatment group remained tumour-free, whereas recurrence occurred 8 days after the last treatment with PEI/MKL1/2- or MKL1 + 2-specific siRNAs in one mouse of either group (Fig 6E).

To prove efficient delivery of MKL1 siRNA into the tumour, a mouse bearing a recurrent tumour was treated three times systemically through i.p. injection of PEI/siRNA MKL1 complexes. Subsequent analysis of the lysate by immunoblotting confirmed that MKL1 protein expression was reduced to 40% (Fig 6F). Similarly, MKL1 and 2 protein expression was strongly diminished in a recurrent tumour treated with PEI-complexed siRNA MKL1/2 (Fig 6F, top). Histological evaluation of mice treated with MKL1/2 siRNA did not reveal any signs of liver damage such as inflammation, necrosis, steatosis or fibrosis (Supporting Information Fig S10).

We then sought to determine whether MKL1 knockdown alone is sufficient to induce cellular senescence *in vivo*. To test this hypothesis, we recultivated HCC tumour cells derived from the recurrent xenograft treated with MKL1 siRNA. We found that these cells entered senescence in culture as determined by staining for senescence-associated β-galactosidase activity (Fig 6G).

We conclude that therapeutic knockdown of MKL1 and 2 in HCC xenografts abolishes HCC xenograft growth by inducing cellular senescence. Moreover, MKL1 knockdown alone is sufficient to abrogate HCC xenograft growth.

## DISCUSSION

HCC is among the most lethal and common cancers in the human population (Farazi & DePinho, 2006). Despite its significance, the molecular mechanisms that drive its progression are not well understood and few molecular targets have been identified for therapeutic intervention.

A major breakthrough towards a targeted therapy of HCC was the discovery that the tumour suppressor DLC1 is deleted in 50% of liver cancers (Xue et al, 2008; Yuan et al, 1998). We recently demonstrated that the transcriptional coactivators MKL1 and 2 mediate the effects of loss of the tumour suppressor DLC1: lack of DLC1 leads to nuclear localization of MKL1/2 in primary human HCC cells, resulting in constitutive activation of several tumour-relevant MKL target genes (Muehlich et al, 2012). Such mechanistic understanding of the genetic basis of HCC prompted us to investigate the effect of MKL1/2 depletion on HCC xenograft growth.

In this paper, we provide the first evidence that depletion of the transcriptional coactivators MKL1 and 2 abolishes tumour growth. Furthermore, we demonstrate that MKL1/2 knockdown can be used as a therapeutic approach in DLC1-deficient HCC tumour xenografts.

**Figure 7:**
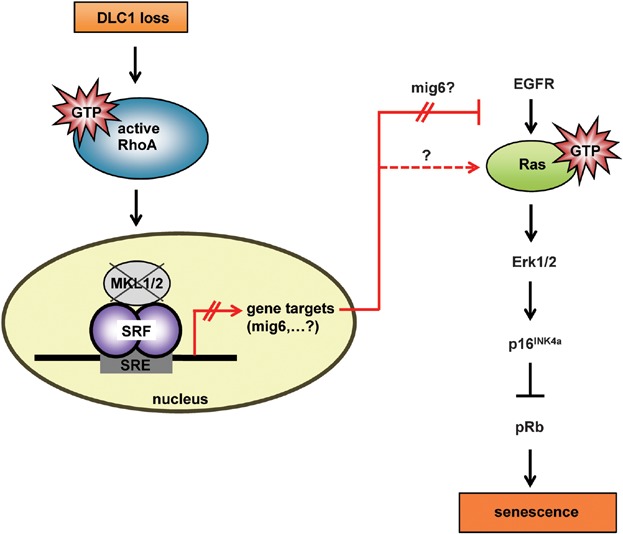
**Proposed model for senescence induction upon MKL1/2 depletion:** DLC1 loss results in RhoA activation and MKL1/2 nuclear localization. Depletion of MKL1/2 induces cellular senescence via activation of the Ras/MAPK and p16^INK4a^/pRb pathways. It is currently unclear how MKL1/2 depletion causes Ras activation, however, impaired expression of MKL target genes such as mig6, a negative regulator of the EGFR-MAPK cascade, could mediate Ras activation.

We proved the *in vivo* efficacy of MKL1 and 2 knockdown in established HCC xenografts by using a PEI-based delivery platform for siRNAs. Our findings open up the possibility that blocking MKL1 and 2 may be harnessed as a novel molecularly targeted therapeutic strategy for the treatment of HCC. On the cellular level, we identified senescence as the mechanism underlying the inhibitory effect of MKL1/2 knockdown on HCC tumour growth. Senescence-associated changes included a flat, vacuole-rich morphology devoid of stress fibres and positive SA-β-Gal activity in MKL1/2-depleted HCC cells. Furthermore, MKL1/2 depletion in HuH7 HCC cells provoked a cell-cycle arrest in the G1 phase, a characteristic feature of cellular senescence. The MKL1/2-mediated senescence response has not been noticed before, probably because the tumour cells used in previous studies express DLC1 (Medjkane et al, 2009). In agreement with this notion, depletion of MKL1/2 in DLC1-expressing HepG2 or HLF cells neither induced senescence, nor affected cell proliferation. HepG2 cells became responsive to the effect of MKL1/2 knockdown on cell proliferation only after depletion of DLC1 expression. Mechanistically, we demonstrate that depletion of MKL1/2 activates the oncogene Ras in DLC1-deficient HCC cells, resulting in increased levels of pERK (ERK^pT202/pY204^) (Fig 7). Descot and colleagues found a similar negative crosstalk between the actin-MKL1 and the MAPK pathway via the MKL target gene mig6 (Descot et al, 2009). Mig6 or other MKL target genes might mediate the effect of MKL1/2 KD on Ras activation. This will be an important question to resolve. We found that the Ras-activated ERK1/2 pathway is responsible for the growth arrest upon MKL1/2 depletion in DLC1-deficient cells, because the MEK1 inhibitor U0126 abolished the anti-proliferative effect of MKL1/2 knockdown. According to a previous study, UO126 suppresses senescence by inhibiting the MEK/mTOR pathway (Demidenko et al, 2009). mTOR might therefore also contribute to the pro-proliferative effect of U0126 in HuH7 MKL1/2 KD cells.

Aberrant activation of the Ras/MAPK pathway by oncogenic Ras has been shown to trigger a senescence response referred to as ‘oncogene-induced senescence’ mediated by the tumour suppressor proteins p16^INK4a^ and p53 (Serrano et al, 1997). In HCC cells, the lack of participation of p53 was expected because HuH7 cells harbor mutant p53 (Hsu et al, 1993). Therefore, we sought to determine whether MKL1/2 depletion elicits up-regulation of p16^INK4a^. Depletion of MKL1/2 strongly induced p16^INK4a^ expression in HuH7 cells treated with MKL1/2 shRNA, whereas p16^INK4a^ was not detectable in HuH7 cells expressing control shRNA.

Interestingly, p16^INK4a^ expression was found to be high in cirrhosis, as compared to normal liver and tumour tissues (Plentz et al, 2007). This observation and the fact that 80% of HCCs are observed in patients with cirrhosis implies that the transition of liver cells into proliferating malignant cells requires the bypass of senescence (El-Serag & Rudolph, 2007). In light of our findings, we propose that MKL1 and 2 contribute to HCC development by enabling this senescence bypass. In accord with this notion, we found that depletion of MKL1 and 2 results in the reversal of the malignant phenotype, characterized by senescence and up-regulation of p16^INK4a^.

It is well known that p16^INK4a^ ultimately activates the retinoblastoma protein Rb which is at the core of senescence because it has been shown to repress transcription of genes necessary for G1-S phase transition and DNA replication mediated by members of the E2F transcription family (Burkhart & Sage, 2008; Chicas et al, 2010). Rb imposes a block on G1 progression that is alleviated by its phosphorylation (Ohtani et al, 2004). Consistent with this, we observed that the G1 arrest in HuH7 MKL1/2 knockdown cells was accompanied by hypophosphorylation of Rb.

Further support for the establishment of oncogene-induced senescence upon MKL1/2 depletion comes from the finding of p53 phosphorylation on serine 15 in HuH7 and HuH6 HCC cells. Di Micco and colleagues have shown that oncogene-induced senescence is a consequence of the activation of a robust DDR, characterized by phosphorylation of p53 on serine 15 (Di Micco et al, 2006). Furthermore, we observed an accumulation of histone H3 methylated on lysine 9 in HuH7 and HuH6 cells. The replacement of histone H3 acetylation by methylation in SAHF is another hallmark of senescence (Narita et al, 2003). The induction of senescence requires several secreted factors, collectively termed the SMS (Kuilman & Peeper, 2009). Amongst the SMS factors, the expression of the chemokine (C-S-C motif) ligand 10 (CXCL10) and TNFSF10 was significantly increased upon MKL1/2 depletion. Both CXCL10 and TNFSF10 have been shown to drive numerous human cancers into senescence (Braumuller et al, 2013). The evaluation of these additional senescence markers *in vitro* and *in vivo* in HuH7 mouse tumour xenografts provides further evidence that depletion of MKL1 and 2 induces senescence.

One of the classical definitions of senescence is that senescent cells are incapable of triggering the expression of genes required for proliferation in response to growth factors (Seshadri & Campisi, 1990). MKL1 and 2 are coactivators of the serum response transcription factor (SRF) that controls cellular processes such as proliferation and migration by transducing growth factor signalling into gene expression. Therefore, MKL1/2 and SRF play a central role in blocking the onset of senescence. In accord with this concept, homozygous inactivation of SRF in murine colon-derived SMCs leads to a senescent phenotype (Angstenberger et al, 2007). However, it should be noted that distinct molecular mechanisms underlie the MKL1/2 and SRF-dependent senescence responses: in contrast to MKL1/2 depletion as investigated here, SRF depletion in primary vascular SMCs did not increase p16^INK4a^ expression (Werth et al, 2010).

One of the most intriguing aspects of our study is that the depletion of MKL1/2 in DLC1-deficient HCC cells and HCC mouse tumour xenografts leads to cellular senescence and thereby mimics the effect of DLC1 re-expression. Senescence induction upon DLC1 restoration has not been described before, possibly due to the use of transient transfections in other studies, not allowing for long-term analysis of cell proliferation and senescence induction (Yuan et al, 2007; Zhang et al, 2009; Zhou et al, 2004). In line with our findings, Zhang and colleagues observed cell cycle arrest in the G_1_ phase upon reintroduction of DLC1 (Yuan et al, 2004; Zhang et al, 2009; Zhou et al, 2004). Since tumour suppressors are generally not amenable to direct therapeutic targeting, pharmacologic intervention at the level of MKL1/2 may have broad therapeutic potential in DLC1-deficient cancers. This is accentuated by our data in mice, where we explored for the first time a therapeutic MKL1 and MKL2 knockdown approach in tumours. We employed polymer-based nanoparticles for the *in vivo* delivery of an siRNA targeting both MKL1 and 2, a combination of MKL1 and MKL2 siRNAs and MKL1 siRNA alone to established HCC xenografts. Polymer-based nanoparticles had been shown to allow for the *in vivo* delivery of siRNAs upon systemic application (Hobel et al, 2010). We chose systemic administration rather than local (intratumoral) injection due to the higher relevance in a therapeutic setting. MKL1/2 knockdown completely abolished HCC tumour growth. We assessed for liver toxicity by histological evaluation of the livers of mice treated with MKL1/2 siRNA. Importantly, no signs of liver damage such as inflammation, necrosis, steatosis or fibrosis were detected.

The combination of MKL1 and 2 siRNA also significantly alleviated tumour growth. The regression of the tumour xenografts was associated with senescence, because p16^Ink4a^ mRNA expression was significantly elevated and the candidate senescence markers, such as accumulation of histone H3 methylated on lysine 9, phosphorylation of p53 at serine 15 and enhanced expression of the senescence-associated chemokine CXCL10 mRNA, were detectable in the tumours of the animals treated with MKL1 and 2 siRNA.

The combination of MKL1 and 2 siRNA proved to be somewhat less efficient as compared to the siRNA targeting both MKL1 and 2. This result may be due to the lower dose of MKL1 and MKL2 siRNAs: the combination of MKL1 and 2 consists of 7.5 μg MKL1 and 7.5 μg MKL2 siRNA, whereas 15 μg MKL1/2 siRNA targeting both MKL1 and 2 was used. It should be noted, however, that comparably low siRNA amounts were used in this animal experiment and proved to be sufficient for tumour abolishment, thus emphasizing the relevance of MKL1/2 and the efficacy of the PEI-based siRNA delivery. It is particularly noteworthy that knockdown of MKL1 alone was sufficient for senescence induction and complete abrogation of HCC tumour formation, and that mice treated with MKL1 siRNA alone remained tumour-free after the end of the therapy and for the whole duration of monitoring (another 4 weeks). In light of the finding that MKL1 knockout mice are viable and fertile, and only fail to nurse their offspring (Li et al, 2006; Sun et al, 2006), we anticipate that a prolonged treatment may not lead to pronounced unwanted side-effects related to other functions of MKL1. Global deletion of MKL2, however, results in embryonic lethality owing to cardiovascular defects (Oh et al, 2005) and may therefore involve more severe unwanted side-effects in a therapeutic setting.

Taken together, our results establish MKL1 and 2 as promising new therapeutic targets for the treatment of HCCs characterized by DLC1 loss. In particular, we envisage that MKL1 inhibitors will have enormous as yet unappreciated therapeutic potential to inhibit the growth of tumours lacking DLC1.

## MATERIALS AND METHODS

### Cell culture, transfections and reagents

HEK293T, HuH7 and HuH6 cells and the amphotropic Phoenix packaging cells were cultured in Dulbecco's modified Eagle's medium (DMEM; Sigma–Aldrich, Taufkirchen) supplemented with 10% fetal bovine serum (FBS; Invitrogen). HepG2 and HLF cells were grown in RPMI 1640 medium with 10% FBS. For the stable knockdown of MKL1 and MKL2, the sequence CATGGAGCTGGTGGAGAAGAA that is common in both human MKL1 and MKL2 was cloned into the pLKO.1 vector as described previously (Lee et al, 2010). Plasmids expressing shRNA targeting MKL1 and MKL2 or control shRNA (nontarget shRNA control vector, SHC002, from Sigma–Aldrich, Taufkirchen) were cotransfected with the packaging plasmids pD8.9 and pVSVG into HEK 293T cells by the calcium phosphate DNA precipitation method as described previously (Sambrook et al, 1989). For the retroviral transduction, amphotropic Phoenix packaging cells were transfected by calcium phosphate DNA precipitation with the pBabe puro H-Ras V12 plasmid. After 48 h, the virus containing supernatant was filtered (0.45 µM filter; Millipore) and supplemented with 4 µg/mL polybrene (Sigma–Aldrich, Taufkirchen). Human HCC cells were infected with the viral supernatant and selected with 5 μg/mL puromycin (Calbiochem, Darmstadt, Germany) for 7 days.

For transient siRNA mediated knockdown, HuH7 cells were reversely transfected with siRNA oligonucleotides (50 nM) using Lipofectamine RNAiMAX (Invitrogen) according to the manufacturer's instructions.

HepG2 cells were transiently transfected using GenJet in Vitro Transfection Reagent (SignaGen Laboratories, Rockville, USA) according to the manufacturer's protocol. For all other transient transfections of human hepatoma cells Lipofectamine 2000 (Invitrogen) was used according to the manufacturer's instructions. The GTP bound state of RhoA and Ras was analyzed by using the G-LISA RhoA Activation Assay Biochem Kit (Cytoskeleton, Denver, CO, USA) and the Ras Activation Assay Kit (NewEast Biosciences, Malvern, USA) according to the manufacturer's instructions.

### Immunoblotting

Cells and tumour samples were incubated in lysis buffer [50 mM HEPES pH 7.4, 150 mM NaCl, 1% Triton X-100, 1 mM EDTA supplemented with protease inhibitors (Calbiochem, Darmstadt, Germany)], homogenized in Laemmli sample buffer and boiled for 10 min. Proteins were resolved by SDS-polyacrylamide gel electrophoresis (SDS-PAGE) and then transferred to polyvinylidene (PVDF) membranes (Millipore, Schwalbach, Germany), which were then incubated with the respective antibodies. The rabbit anti-MKL1 antibody was generated by injecting rabbit with glutathione S-transferase (GST)-MKL1 (amino acids 601–931) purified from *Escherichia coli*. Antiserum to MKL2 was generated by injecting rabbits with GST-MKL2 (amino acids 703–1049) purified from *E. coli* as described by Cen et al (Cen et al, 2003).

The primary antibodies used were mouse anti-p16^INK4a^ (MTM Laboratories, Heidelberg, Germany), rabbit anti-Retinoblastoma protein (Clone EP44) (Epitomics, California, USA), rabbit anti-phospho-p44/42 MAPK (Erk1/2) (Thr202/Tyr204) (Cell Signaling Technology, Danvers, USA), mouse anti-SMA Clone 1A4 (Dako, Hamburg, Germany), rabbit anti-SM22 (GeneTex, Irvine CA, USA), rabbit anti-Erk C14 (Santa Cruz Biotechnology, CA, USA), mouse anti-DLC1 (BD Biosciences, Heidelberg, Germany), rabbit anti-H3K9me3 (Actif Motif, Carlsbad, USA), mouse anti-p53 (Clone DO1) (Actif Motif, Carlsbad, USA), rabbit anti-phospho p53 Serine 15 (Santa Cruz Biotechnology, CA, USA), mouse anti-Hsp90 (Santa Cruz Biotechnology, CA, USA), rabbit anti-RhoA (NewEast Biosciences, Malvern, USA). Bound antibodies were detected using enhanced chemiluminescence at a luminescent imager (Peqlab, Erlangen, Germany).

### Senescence-associated β-Galactosidase staining

Cellular senescence was determined by using the senescence β-galactosidase staining kit according to the manufacturer's instructions (Cell Signaling Technology, Danvers, USA). By counting the number of the cells with blue colour and the total cells per field (0.5 × 0.5 cm^2^) under an inverted Zeiss microscope, the percentage of the SA-β-gal-positive cells was calculated. More than 1000 cells were counted from four fields and presented as mean ± standard deviation.

**The paper explained**

**PROBLEM:**

Hepatocellular carcinoma (HCC) is among the most lethal and common cancers in the human population. Despite its significance, few molecular targets have been identified for therapeutic intervention. The tumour suppressor deleted in liver cancer 1 (DLC1) is lost in approximately 50% of liver cancers. Our lab has recently shown that the transcriptional coactivators MKL1 and 2 mediate the tumorigenic effects upon DLC1 loss. The aim of this work was to assess whether MKL1/2 could constitute a new therapeutic target in DLC1-deficient HCC.

**RESULTS:**

Here, we report that depletion of MKL1 and 2 inhibits tumour growth of DLC1-deficient HCC cells by inducing oncogene-induced senescence. Restoration of DLC1 expression in HCC cells also induced cellular senescence, thereby mimicking the effect of MKL1/2 depletion. To assess the therapeutic efficacy of MKL1/2 knockdown *in vivo*, we treated nude mice bearing HCC xenografts systemically with MKL1/2 siRNAs using polyethylenimine (PEI) complexation as an efficient tool for *in vivo* siRNA delivery. We found that therapeutic knockdown of MKL1 and 2 completely abolished HCC xenograft growth. Importantly, MKL1 siRNA alone was sufficient for complete abrogation of HCC xenograft growth.

**IMPACT:**

This is the first evidence that MKL1 and 2 are promising novel therapeutic targets for the treatment of HCC characterized by DLC1 loss. Since tumour suppressors such as DLC1 are generally not amenable to direct therapeutic targeting, we envisage that pharmacologically targeting MKL1/2 will have enormous potential as a personalized medicine approach for DLC1-deficient cancers.

### RNA extraction, cDNA synthesis and quantitative real time PCR analysis

Total RNA was isolated from human hepatoma cell lines using TRIzol reagent according to the manufacturer's instructions (Invitrogen).

RNA (1 µg) was primed with random hexamers and reverse transcribed into cDNA with SuperScript II Reverse Transcriptase (Invitrogen). PCR reactions were performed using the LightCycler 480 Real-Time PCR System (Roche, Mannheim, Germany). MRNA expression was normalized to the endogenous housekeeping control gene 18S rRNA.

### Cell proliferation assay

1 × 10^5^ human HCC cells were seeded in 6 wells and harvested at 24 h intervals to count the cell number with the hematocytometer.

### FACS analysis

Cells were harvested, washed with PBS and resuspended in a staining solution containing 10 mM sodium chloride, 3.8 mM trisodium citrate and 0.3 mL/L Nonidet P-40 supplemented mit propidium iodide. To avoid unspecific binding of propidium iodide to RNA, cells were treated with RNase (Fermentas). After 60 min incubation at room temperature, a second staining solution containing 70 mM citric acid and 0.25 M saccharose supplemented with propidium iodide was added.

After further 30 min incubation, stained cells were analysed by flow cytometry (BD FACSCalibur Flow Cytometer, New Jersey, USA).

### Immunostaining

Cells were fixed with 4% paraformaldehyde in PBS for 10 min at room temperature and then permeabilized with 0.2% Triton X-100 in PBS for 7 min. Blocking of unspecific binding sites was performed by incubation in 1% bovine serum albumin in PBS for 60 min at room temperature. Incubations with the primary antibodies goat anti-MRTF-A (C-19) (Santa Cruz Biotechnology, CA, USA), mouse anti-FLAG M2 (Sigma–Aldrich, Taufkirchen, Germany), anti-active caspase-3 (Promega, Mannheim, Germany) were carried out for 60 min followed by the incubation with the secondary antibodies labelled with the Alexa Fluor 488/555 dye (Invitrogen) for 30 min. Cells were washed four times with PBS after antibody incubations and before mounting. F-actin filaments were stained with phalloidin coupled to Alexa Fluor 488 (Invitrogen) and nuclei were stained with 1 µg/mL 4′6-diamidion-2-phenylindole (DAPI) (Sigma–Aldrich, Taufkirchen). Images were obtained using a confocal microscope (Zeiss).

### Subcutaneous tumour xenograft model in nude mice

Effects of RNAi mediated knockdown of MKL1 and MKL2 on HuH7 tumour xenograft growth *in vivo* were determined by systemic injection of siRNA complexed with PEI as described previously (Hobel et al, 2010). Athymic nude mice were held under specific pathogen-free conditions with ad libitum access to food and water. Experiments were approved by the government of Upper Bavaria and performed according to legal terms for animal experiments of the local administration. 2 × 10^6^ HuH7 cells were injected subcutaneously into both flanks of 6 week old female athymic nude mice (Crl:CD1-Foxn1nu, Charles River Laboratories, Sulzfeld, Germany). After 7 days, when solid tumours were established, mice were randomized into control and treatment groups with 6 animals per group. Mice were intraperitoneally injected three times per week with 15 µg nonspecific or specific siRNAs, each complexed with PEI F25-LMW. The following siRNA sequences were used:

siRNA Neg. ctrl 5′-CGUACGCGGAAUACUUCGAdTdT-3′;
siRNA MKL1/2 5′-AUGGAGCUGGUGGAGAAGAAdTdT-3′;
siRNA MKL1 5′-GAAUGUGCUACAGUUGAAAdTdT-3′;
siRNA MKL2 5′-GUAACAGUGGGAAUUCAGCdTdT-3′.

For the siRNA MKL1 + 2 treatment group, 7.5 µg siRNA MKL1 and 7.5 µg siRNA MKL2 were mixed and complexed with PEI L25-LMW. Untreated mice or mice treated with nonspecific siRNA served as negative control. Tumour volume was measured three times per week and calculated according the formula length × width × width/2. Mice bearing subcutaneous tumours were sacrificed on day 22 after therapy start, tumours excised and shock frozen in liquid nitrogen for subsequent RNA and protein preparation. For histological evaluation tumours were formalin fixed, paraffin embedded, sectioned at 5 µm and stained with hematoxylin and eosin. Sections were evaluated by a pathologist and mitotic counts were performed in five 40× high power fields (HPFs). Mice without tumours were kept alive to check for the recurrence of tumours for another 4 weeks. Mice bearing recurrent tumours were retreated again three times with siRNA/PEI complexes and then sacrificed for subsequent protein and RNA preparation of the tumours as described above. Additionally, a part of the tumour was trypsinized with 0.05% Trypsin-EDTA and resuspended in DMEM medium to culture cells derived from the xenografts *in vitro* for senescence-associated β-galactosidase staining.

### Statistical analysis

The statistical analysis was performed using the Student's-*t*-test. Unless otherwise indicated, the values are presented as mean ± SD.

## Supplementary material

As a service to our authors and readers, this journal provides supporting information supplied by the authors. Such materials are peer reviewed and may be re-organized for online delivery, but are not copy-edited or typeset. Technical support issues arising from supporting information (other than missing files) should be addressed to the authors.
