# Supplementary material for: Depletion of the transcriptional coactivators megakaryoblastic leukaemia 1 and 2 abolishes hepatocellular carcinoma xenograft growth by inducing oncogene-induced senescence
Source: EMBO Mol Med. 2013 Jul 29;5(9):1367–82. doi: 10.1002/emmm.201202406 (PMC3799492; doi:10.1002/emmm.201202406)
Supplement: Supplementary file 1 [file emmm0005-1367-SD1.pdf]

EMBO
Molecular Medicine

Depletion of the transcriptional coactivators Megakaryoblastic Leukemia 1 and 2 abolishes hepatocellular carcinoma xenograft growth by inducing oncogene-induced senescence

Veronika Hampl, Claudia Martin, Achim Aigner, Sabrina Hoebel, Stephan Singer, Natalie Frank, Antonio Sarikas, Oliver Ebert, Ron Prywes, Thomas Gudermann and Susanne Muehlich

Corresponding author: Susanne Muehlich, Ludwig-Maximilians-University Munich

Review timeline:

Submission date:	19 December 2012
Editorial Decision:	12 February 2013
Revision received:	21 May 2013
Editorial Decision:	11 June 2013
Revision received:	17 June 2013
Editorial Decision	20 June 2013
Additional Correspondence (author)	21 June 2013
Accepted:	21 June 2013

Transaction Report:

(Note: With the exception of the correction of typographical or spelling errors that could be a source of ambiguity, letters and reports are not edited. The original formatting of letters and referee reports may not be reflected in this compilation.)

Editor: Roberto Buccione

1st Editorial Decision 12 February 2013

Thank you for the submission of your manuscript to EMBO Molecular Medicine. We are sorry for the delay in getting back to you with the Reviewers' evaluation on your work. Unfortunately, we experienced difficulties in securing three appropriate Reviewers and then obtaining their evaluations in a timely manner. We have now heard back from the three Reviewers whom we asked to evaluate your manuscript.

You will see that all three are generally supportive of you work but raise significant issues that question the conclusiveness of the results thus preventing us from considering publication at this time. I will not dwell into much detail, as the evaluations are detailed and self-explanatory. I would like, however, to highlight a few main points.

Reviewer 1 has two main areas of concern. On one side s/he points to lack of sufficient mechanistic insight with respect to how MKL1/2 knockdown leads to Ras/Erk activation, and would like to know whether DLC1 knockdown renders the cells responsive to the effects of MKL1/2 knockdown and if the regression of xenografts is associated with tumour senescence. The latter two are important points that need to be clarified experimentally. As for the first, I would simply encourage developing the study as far as realistically possible to strengthen your findings. The second, fundamental point raised by Reviewer 1 pertains to the need of an appropriate animal model of hepatocellular carcinoma (HCC) to reach meaningful conclusions. This is an important aspect for EMBO Molecular Medicine and thus I strongly suggest that you take advantage of the many existing mouse models of HCC to strengthen the clinical (and overall) impact of your findings. Reviewer 1 notes additional issues that require your action.

Reviewer 2 is especially concerned that the experimental demonstration of oncogene-induce senescence in vitro and in vivo is not sufficiently tight and thus additional experimentation is required to this effect. This Reviewer also lists other critical points that require your intervention.

Reviewer 3 is concerned that the xenograft experiments with DLC1-deificient HCC cells lack an important control. S/he also provides a detailed list of experimental shortcomings and suggests a number of improvements that you should act upon to increase the overall quality of data and presentation.

Considered all the above, while publication of the paper cannot be considered at this stage, we would be prepared to consider a suitably revised submission, with the understanding that the Reviewers' concerns must be fully addressed with additional experimental data where appropriate and that acceptance of the manuscript will entail a second round of review.

Since the required revision in this case appears to require a significant amount of time, additional work and experimentation and might be technically challenging, I would therefore understand if you chose to rather seek publication elsewhere at this stage. Should you do so, we would welcome a message to this effect.
Please note that it is EMBO Molecular Medicine policy to allow a single round of revision only and that, therefore, acceptance or rejection of the manuscript will depend on the completeness of your responses included in the next, final version of the manuscript.

As you know, EMBO Molecular Medicine has a "scooping protection" policy, whereby similar findings that are published by others during review or revision are not a criterion for rejection. However, I do ask you to get in touch with us after three months if you have not completed your revision, to update us on the status. Please also contact us as soon as possible if similar work is published elsewhere.

I look forward to seeing a revised form of your manuscript as soon as possible.

***** Reviewer's comments *****

Referee #1 (Remarks):

Hampl et al. describe the interesting finding that two established HCC cell lines (HuH6 and HuH7), carrying deletion of the DLC1 gene, display (i) elevated activity of Rho (previous paper by the authors), (ii) impaired proliferation upon knock-down of MKL1/2, (iii) elevated activation of Ras/Erk upon knock-down of MKL1/2, (iv) elevated senescence upon knock-down of MKL1/2, and (v) impaired HuH7 tumor xenografting efficiency upon in vivo MKL1/2 knock-down. Two different HCC cell lines (HepG2, HLF), which have wt DLC1, do not show the above effects. The important suggestion is derived that DLC1-negative cancers might be treated successfully by strategies involving downregulation of MKL1/2.

The experiments are performed competently and the generated results are convincing.

The criticism of this reviewer concerns two major areas, which should be improved and strengthened before the manuscript can be considered worthy for EMM.
A. The manuscript lacks functional insight and leaves open important questions:
• How does MKL1/2 knockdown cause activation of Ras/Erk?
• Does knockdown of DLC1 expression in HepG2 cells render these cells responsive to MKL1/2 knockdown effects? How much of the observed effects are a consequence of established cell lines having accumulated other functional characteristics.
• Is regression of xenografts associated with senescence in the tumors of the animals?
B. Overinterpretation of the xenograft studies.
Subcutaneous tumor xenografts are not HCCs! The last sentence of the Results section exemplifies this misconception and is extremely misleading: 'Moreover, MKL1 knockdown alone is sufficient to abrogate HCC tumor growth ......'. To reach more meaningful conclusions, a better animal model should be employed, which reflects the biology of HCC.

copyright EMBO

Minor points:
• Introduction: '.....however, the functional role of MKL1/2 in tumors has not been studied......'. This statement completely neglects to observations of MKL fusion proteins being causative to some leukemia formation.

Results:
• Figure 1A: There is some residual DLC1 signal in lane 1 (HuH7). Is this an artifact of this gel or is there some true DLC1 expression?

Materials and Methods
• The origin (supplier) and specificities of the anti-MKL1 and anti-MKL2 antibodies are not revealed.

Referee #2 (Comments on Novelty/Model System):

-Technical quality : incomplete for senescence analysis
-novelty : high but the conclusion "OIS induction" is only patially supported by the data
-adequacy of the model system: the in vitro and in vivo cellular systems are adequate to look at MLK1/2 depletion effects in hepatocellular carcinoma

Referee #2 (Remarks):

In this study, the authors were interested in studying the effects of abolishing MKL1/2 over hepatocellular carcinoma growth. They observed that depletion of MKL1/2 provokes proliferation arrest in hepatocellular carcinoma cells with DLC1 loss. They next involved RhoA activity and subsequent oncogene-induced senescence (OIS) in MKL1/2 depletion effects and they next displayed some data showing the therapeutic effect of MKL1/2 knockdown in vivo. The authors conclude that MKL1/2 depletion, in a context of DLC1 depleted cells, blocks hepatocellular carcinoma growth through OIS induction.

Major points:
Whereas the effects of MKL1/2 knockdown in inhibiting cancer cell growth in vitro and in vivo are well demonstrated, the detection of OIS is not performed adequately. SAbGal assay absence is sufficient to conclude that there is no senescence but its presence is not sufficient to conclude that there is senescence. Other senescence markers are required such as components of the senescence associated secretome (IL8...) and/or DNA damage or its signalling induction. This is required in Figure 4 to conclude that MLK1/2 KD cells are in senescence.
For the in vivo experiments (Figure 6), the number of SAbGal positive cell is of 2% in the ctrl vs 4% siMKL1. This differential is too low to conclude that there is an increased SAbGal activity that might explain the phenotype, in addition this requires other(s) senescence markers.

Other points:
-Figure 2: nuclear counterstaining is required to conclude about MKL1 localization.

-Figure 4: Ras and its downstream targets activation (Raf, Mek...) are well known inducers of OIS in "normal" primary cells. Its ability to induce OIS in cancer cells is not the rule and is even probably the exception. It will be nice to show that constitutive expression of an active Ras or/and downstream effecters (Raf or Mek) are able to induce OIS in HuH6 and 7 cells.
Efficiency of UO126 treatment should be checked by performing immunoblot against phospho-ERK in MKL1/2 KD cells.
Involvement of p16 should also be check by performing its knockdown in MLK1/2 KD cells.

-Figure 5: to claim that adding DLC1 in DLC1-negative cells induces senescence, the authors should check that the cells stop to proliferate by performing growth curves experiments and display at least one other senescence markers. HuH7DLC1 without dox cells should also be compared to HuHDLC1 cells with dox and not only with HuH7.

-Figure 6: tumor volume (6C) include various conditions (siMKL1/2, siMKL1+2, siMKL1) but the experiments around of validation of the tools or analysis were not performed with the 3 conditions. The rationale for this is difficult to understand.
Fig 6C, siMKL1 display a better efficiency to inhibit tumor growth than siMKL1/2 or siMKL1+2, the author should comment and discuss this result and this might mean that for the all in vitro experiments inhibiting MKL1 and 2 was useless and the experiments should have been performed only with MKL1 KD.

Referee #3 (Comments on Novelty/Model System):

One of the main novel findings is that tumors, formed in mice that were xenografted with a HCC cell line in which DLC1 has been deleted, regress after systemic delivery of siRNA's that target MKL1. This is cool. But, they do not use a control cell line that doesn't have mutant DLC1. The best control would be the HCC line that does not have a DLC1 mutation, but into which they put a DLC1 mutation as in Figure 2. After DLC1 is mutated, then this cell line also is inhibted by MKL1 siRNA. This control - or another nonresponsive cell line control would enrich the model.

Referee #3 (Remarks):

Hampl,...Muehlich
Depletion of the transcriptional coactivators MKL1 and MKL2 abolishes hepatocellular carcinoma growth by inducing oncogene-induced senescence

In this manuscript, the authors focus on the role of the MRTF family (MKL1 and MKL2) of co-transcriptional activators in DLC1-deficient hepatocellular carcinoma (HCC), particularly with regards to tumor growth. DLC1 is a tumor suppressor with RhoGAP activity. A previous publication (Oncogene 2012) from this group demonstrated that MKL1/2 signaling is required to promote transformation in DLC1-deficient HCC, potentially via the constitutive nuclear localization of these factors downstream of enhanced RhoA activity. In this previous paper, they showed that MKL1/2 were required for cell proliferation upon depletion of DLC1 in HuH7 cells and MDA-MB cells. In addition, they showed that RhoA activation inhibited ERK1 activation, thereby preventing MKL1 inactivation by phosphorylation.

In the submitted manuscript, the investigators confirm that knockdown of MKL1/2 in DLC1-deficient HCC cell lines impairs cell proliferation, but has no effect on growth of DLC1-positive HCC cells, suggesting that DLC1 expression status is important in predicting tumor dependence on MKL1/2. Again, they show that this effect on proliferation is dependent on active RhoA. Expression of constitutively active RhoA with MKL1/2 knockdown in a DLC1-positive cell line mimicked the growth inhibition of DLC1-deficient cells with MKL1/2 knockdown. What was new in this manuscript was that the authors determined that the growth inhibition was not via apoptosis, but via cell cycle arrest and senescence. MKL1/2 knockdown in DLC1-deficient cells increased Ras activation, ERK1/2 phosphorylation, p16 expression, and hypo-phosphorylation of Rb. Together with observed morphological changes, the authors concluded that there was evidence of oncogene-induced senescence, with growth arrest via Ras/ERK signaling. Consistent with this, enforced expression of DLC1 in a DLC1-deficient cell line also promoted senescence and G1 arrest, suggesting that DLC1 restoration could mimic MKL1/2 knockdown. Given these observations, the authors hypothesize that modulation of MKL1/2 signaling will affect the growth behavior of this particular tumor type in vivo. They test this hypothesis using siRNAs in a HCC xenograft model using the same DLC1-deficient HCC cell lines. MKL1/2 knockdown significantly impaired tumor growth, but only MKL1 knockdown prevented tumor recurrence. In addition, MKL1 knockdown was sufficient to impair tumor growth via induction of senescence. Based on data from this single xenograft model, the authors believe that MKL1 modulation represents a promising therapeutic strategy to treat DLC1-deficient HCC.

The major strengths of this paper:
1. The findings provide further support of their mechanistic explanation linking MKL1/2 and growth of solid tumors
2. One of the first demonstrations of the therapeutic potential of targeting MKL1/2

Major Concerns:

1. There is a lack of experimental detail, particularly with regard to the 'n' for the experiments shown. This should be clarified throughout and all experiments showing statistical significance need to have been performed a minimum of 2 times.
2. In Figure 1, the investigators should show a western blot of the cell lines expressing roughly equivalent amounts of MKL1/2. Also is Fi 1, better labels are needed on figures (e.g. For Fig 1B, under controls maybe put Scr; for Fig 1E, mark as DLC1-deficient cell lines, and for 1F, indicate that these are DLC1+ cell lines.
3. For MKL1 mRNA expression, what is it relative to (what exactly is the y-axis)? The control in the SM22 graph is 1, which makes sense, but the controls in the MKL1 graphs are not 1.
4. If HepG2 and HLF do not have nuclear MKL1, then they have no active MKL1 to inhibit. This should be clarifies for the readers.
5. For the data shown in Figure 2, why did you use a Tet-off system if you are not taking advantage of this? (e.g. performing experiments on identical calls + and - dox and show that MKL1 returns to cytoplasm? DAPI staining should be shown for Fig 2B. For Fig 2D, the investigators should show controls without active RhoA. Is the active RhoA affecting cell growth compared to controls? Also, is ther partial rescue of HuH6/7 + MKL1/2 KD + RhoAV14?
6. For the data shown in Figure 4, HepG2 should also be quantified in Fig 4A. Also, the authors should label IP and IB. Also, the authors should indicate and discuss that U0126 can also affect mTOR.
7. For the data shown in Figure 5, does re-expression of DLC1 overcome MKL1/2 KD and prevent the anti-proliferative effect of KD?
8. For the data shown in Figure 6, an excellent control would be HepG2 xenografts not responding to the MKL1/2 KD.

Minor concerns:
1. Why were some standard techniques not used - GTPase pull down, Ki67?
2. What is the prognostic significance of DLC1+ versus deficient HCC - can prognosis be subclassified based on DLC1 or MKL1/2 expression/dependence?
3. What about the MKL1 inhibitor CCG-1423? Can this be used in early stage cirrhotic lesions to prevent MKL1/2 nuclear localization, and therefore prevent progression to HCC?
4. Knockdown of MKL1 systemically as a therapeutic strategy might have systemic effects. Minimally, the investigators should indicate what toxicities they assessed for, and the discussion should include potential side effects of systemically blocking MLK1 and 2 (e.g. platelet counts, hepatocyte maturation)
5. How might these results link with the role of MKL1 in leukemia?
6. Does DLC1 deficiency affect Ras activity? Is Ras mutated in any of these cell lines employed?
7. For completeness, Fig 1 should include MKL1 mRNA for the HuH6 cell line.
8. In Figure 3, the authors need to clarify what day after MKL1/2 KD the studies were performed.
9. In figure 6D, why were other siRNA conbinations not shown and quantitated? How were the 5 40x fields per sample collected? What is the n? Why is there no error bar for the third column

1st Revision - authors' response 21 May 2013

Referee #1: Major points:

1. How does MKL1/2 knockdown cause activation of Ras/Erk?

<u>Response</u>:

We thank the reviewer for bringing up this important question. In an attempt to answer this question, we tested whether MKL1/2 knockdown might affect RhoA activity. Morin and colleagues have reported an inverse correlation between RhoA and Erk activity, e.g. reduced Erk activation in cells

expressing active RhoA (Morin et al, 2009). To test whether MKL1/2 knockdown could cause activation of Ras/Erk by decreasing RhoA activity, we assessed the RhoA activation status by using a Rho G-LISA assay as described previously (Muehlich et al, 2012). However, there was no decrease in RhoA activity in HuH7 MKL1/2 KD cells as compared to HuH7 control cells (Fig. R1, this rebuttal).

We are currently testing the hypothesis whether MKL1/2 target genes might mediate the effects of MKL1/2 KD on Ras/Erk activation. Consistent with this hypothesis, Descot and colleagues found a negative crosstalk between the actin-MKL1 and the MAPK pathway via the MKL target gene mig6 (Descot et al, 2009). However, we believe that a detailed analysis of such a crosstalk is beyond the scope of the present paper.

However, in order to address the issue raised by the reviewer in the manuscript, we added the following sentences on page 16:

Descot and colleagues found a similar negative crosstalk between the actin-MKL1 and the MAPK pathway via the MKL target gene mig6 (Descot et al, 2009). Mig6 or other MKL target genes might mediate the effect of MKL1/2 KD on Ras activation. This will be an important question to resolve.

2. Does knockdown of DLC1 expression in HepG2 cells render these cells responsive to MKL1/2 knockdown effects? How much of the observed effects are a consequence of established cell lines having accumulated other functional characteristics.

Response:

To address the critique of Reviewer 1, an shRNA vector targeting DLC1 was introduced in HepG2 cells, resulting in a knockdown efficiency of 83% on DLC1 protein level (revised Fig. 1G). We observed enhanced cell proliferation in HepG2 cells expressing DLC1 shRNA as compared to control shRNA populations. Knockdown of MKL1/2 in HepG2 DLC1 shRNA expressing cells abolished the pro-proliferative effect of DLC1 knockdown (revised Fig. 1H) and induced SA-ß-Gal activity (revised Fig. 3D), strongly suggesting that DLC1 deficiency renders these cells responsive to the effect of MKL1/2 knockdown on cell proliferation. To ultimately rule out the possibility that HCC cells might have accumulated other functional characteristics, we introduced DLC1 and MKL1/2 shRNAs into hepatocytes by lentiviral transduction. Knockdown efficiencies of DLC1 and MKL1/2 were > 80 %, respectively (Fig. R2, this rebuttal). Consistent with the obtained data in HepG2 cells, MKL1/2 knockdown led to a strong decrease in cell proliferation (Fig. R3, this rebuttal). Furthermore, phosphorylation of ERK1/2 and p16 expression was strongly increased upon MKL1/2 knockdown, indicating that oncogene-induced senescence is the mechanism underlying the inhibitory effect of MKL1/2 on cell proliferation (Fig. R4, this rebuttal). Taken together, these data demonstrate that knockdown of DLC1 renders the cells responsive to the effects of MKL1/2 siRNA on cell proliferation.

We added the following section on page 6 (results), page 8 (results) and on page 15 (discussion):

copyright EMBO

Results page 6:
To ultimately test whether DLC1 deficiency plays a causal role in the MKL1/2 knockdown-mediated growth arrest, an shRNA vector targeting DLC1 was introduced in HepG2 cells, resulting in a knockdown efficiency of 83% on DLC1 protein levels (Fig. 1 G). We observed enhanced cell proliferation in HepG2 cells expressing DLC1 shRNA as compared to control shRNA populations. Knockdown of MKL1/2 in HepG2 DLC1 shRNA expressing cells abolished the pro-proliferative effect of DLC1 knockdown (Fig. 1H), demonstrating that DLC1 deficiency renders HepG2 cells responsive to the effect of MKL1/2 knockdown on cell proliferation.

Results page 8:
Positive SA-ß-gal activity upon MKL1/2 knockdown was detectable only after depletion of DLC1 expression in HepG2 DLC1/MKL1/2 double knockdown cells.

Discussion page 15:
HepG2 cells became responsive to the effect of MKL1/2 knockdown on cell proliferation only after depletion of DLC1 expression.

3. Is regression of xenografts associated with senescence in the tumors of the animals?

Response: To provide evidence that regression of xenografts is associated with senescence, we directly determined $p16^{INK4a}$ expression, the hallmark of senescent cells (Michaloglou et al, 2005). $P16^{INK4a}$ mRNA expression was significantly elevated in tumours of mice treated with MKL1+2 siRNAs. We were able to verify the other candidate senescence markers from Fig. 4 in vivo, such as phosphorylation of p53 at Ser15 and an accumulation of histone H3 methylated on lysine 9 (H3K9me3). CXCL10 mRNA as a component from the senescence-associated secretome, was also strongly enhanced in the MKL1+2-depleted tumours. Furthermore, we assayed the tumours for the presence of cells that are actively proliferating by measuring Ki-67 levels, and found a strongly reduced Ki-67 mRNA expression in the tumours. Taken together, these data demonstrate that the regression of the xenografts is associated with senescence.
We have revised Fig. 6 accordingly, inserted new Fig. 6 C (Ki-67 expression) and D (p16, H3K9me3, p53Ser15 and CXCL10 expression) and added this text on page 13 (results) and page 19 (discussion):

Results page 13:
In order to confirm that the regression of the xenografts is associated with senescence in the tumors treated with MKL1+2 siRNA, we determined $p16^{INK4a}$ expression. $P16^{INK4a}$ mRNA expression was significantly elevated in tumors of mice treated with MKL1+2 siRNA. Furthermore, we were able to verify the other candidate senescence markers shown in Fig. 4 in vivo, such as an accumulation of histone H3 methylated on lysine 3 (H3K9me3), phosphorylation of p53 at serine 15 and enhanced CXCL10 expression as a component of the senescence-associated secretome (Fig. 6D).

Discussion page 19:

The regression of the tumor xenografts was associated with senescence, because $p16^{Ink4a}$ mRNA expression was significantly elevated and the candidate senescence markers, such as accumulation of histone H3 methylated on lysine 9, phosphorylation of p53 at serine 15 and enhanced expression of the senescence-associated chemokine CXCL10 mRNA, were detectable in the tumors of the animals treated with MKL1 and 2 siRNA.

B. Overinterpretation of the xenograft studies.

Subcutaneous tumor xenografts are not HCCs! The last sentence of the Results section exemplifies this misconception and is extremely misleading: 'Moreover, MKL1 knockdown alone is sufficient to abrogate HCC tumor growth….'.

To reach more meaningful conclusions, a better animal model should be employed, which reflects the biology of HCC.

Response:

We thank reviewer 1 for his suggestion to employ a better animal model. Unfortunately we are not able to take advantage of the mouse models of HCC, because murine HCCs don't contain DLC1 deletions (Xue et al, 2008). In contrast, DLC1 is deleted in 50% of liver, breast, lung and 70% of colon cancers, implying an important role of DLC1 loss in human cancer (Lahoz & Hall, 2008).

Based on our findings, DLC1 loss is the prerequisite for the effect of MKL1/2 depletion on HCC cell proliferation. The latter two reasons made the xenograft mouse model using human HCC cells ideally suited to assess the therapeutic efficacy of MKL1/2 knockdown in vivo.

In an attempt to address the critique of reviewer 1, we set out to establish a rat HCC model. In this model, syngeneic McA-RH7777 rat HCC cells are infused via the portal vein to establish multifocal HCC lesions within the liver (Shinozaki et al, 2004). Characterization of the rat HCC cells revealed, however, that they express DLC1 and exhibit cytoplasmic MKL1 localization (Fig. R5 and R6, this rebuttal). Based on our finding of an essential role of DLC1 loss and concomitant MKL1 nuclear localization for HCC cell proliferation, we couldn't use the rat HCC model. We conclude that DLC1 loss is a characteristic feature of human HCCs that doesn't occur in mice and rats. For these reasons, we chose the xenograft model using human HCC cells. We refrained from implanting the HCC cells directly into the livers of the nude mice, because the delivery of the PEI/siRNA complexes to the liver has not been tested before and performing pharmacokinetics studies on the delivery of the PEI/siRNA complexes is beyond the scope of this study. The complexation of the siRNA has been optimized for subcutaneous tumor xenografts. We agree with the reviewer that these subcutaneous tumor xenografts are not bona fide HCCs and refer to as "HCC xenografts" throughout the paper on the following pages:

- page 1: title
- page 5: last sentence introduction
- page 14: last sentence results

- page 15: discussion sentence 1: HCC xenograft growth
- page 15: discussion sentence 3: HCC tumor xenografts
- page 28: last sentence results (The paper explained): HCC xenograft growth

Referee #1: Minor points:

1. Introduction: '.....however, the functional role of MKL1/2 in tumors has not been studied......'. This statement completely neglects to observations of MKL fusion proteins being causative to some leukemia formation.

Response:

We are grateful to Reviewer 1 for pointing out that information about the MKL1 fusion protein in megakaryoblastic leukemia is missing.

We added the following sentence in the introduction on page 4:
This is reminiscent of the MKL1 fusion protein with the RBM15 gene (a.k.a. OTT), which is a translocation found in acute megakaryoblastic leukemia. RBM15-MKL1 is constitutively nuclear and activates SRF and its target genes (Descot et al, 2008; Ma et al, 2001; Mercher et al, 2001).

2. Results: There is some residual DLC1 signal in lane 1 (HuH7). Is this an artifact of this gel or is there some true DLC1 expression?

Response:
We apologize for the artifact of the gel. We inserted a clean western blot displaying the DLC1 protein expression in revised Fig. 1A.

3. Material and Methods: The origin (supplier) and specificities of the anti-MKL1 and anti-MKL2 antibodies are not revealed.

Response:
The rabbit anti-MKL1 serum was generated by injecting rabbits with glutathione S-transferase (GST)-MKL1 (amino acids 601 to 931) purified from *Escherichia coli.* Antiserum to MKL2 was generated by injecting rabbits with GST-MKL2 (amino acids 703 to 1049) purified from *E.coli*, as described by Cen et al. (Cen et al, 2003). We apologize for the lack of information about the anti-MKL1 and anti-MKL2 antibodies and added the two aforementioned sentences on page 18.

Referee #2: Major points:

1. Whereas the effects of MKL1/2 knockdown in inhibiting cancer cell growth in vitro and in vivo are well demonstrated, the detection of OIS is not performed adequately. SAbGal assay absence is sufficient to conclude that there is no senescence but its presence is not sufficient to conclude that there is senescence. Other senescence markers are required such as components of the senescence associated secretome (IL8...) and/or DNA damage or its signalling induction. This is required in Figure 4 to conclude that MLK1/2 KD cells are in senescence.

Response: To further corroborate that depletion of MKL1 and 2 results in oncogene-induced senescence, we evaluated additional senescence markers.
DNA damage: Di Micco and colleagues have shown that oncogene-induced senescence is a consequence of the activation of a robust DNA damage response, characterized by p53 phosphorylation on S15 (Di Micco et al, 2006). p53 Ser15 phosphorylation was observed in HuH7 and HuH6 MKL1/2 KD cells (new Fig. 4F), but not in HepG2 MKL1/2 KD cells, further supporting the establishment of oncogene-induced senescence in these cells.
Senescence-associated heterochromatic foci (SAHFs): Senescence is associated with global changes in chromatin structure, leading to a replacement of histone H3 acetylation by methylation at lysine 9 in senescence-associated heterochromatin foci (SAHFs) (Narita et al, 2003). We observed an accumulation of histone H3 methylated on lysine 9 (H3K9me3) in HuH7 and HuH6 MKL1/2 KD, but not in HepG2 MKL1/2 KD cells, confirming that MKL1/2 KD cells are in senescence (new Fig. 4G).
Senescence-associated secretome (SAS): The induction of senescence requires several secreted factors, collectively termed as "senescence-messaging secretome" (Kuilman and Peeper, Nature Reviews Cancer 2009). To identify SMS factors, we performed a microarray in HuH7 and HuH7 MKL1/2 KD cells. We found a profound increase in chemokine (C-S-C motif) ligand 10 (CXCL10) and TNFSF10 mRNA expression in HuH7 MKL1/2 KD cells, which has previously been reported as being secreted by senescent cells (Braumuller et al, 2013; Cahu et al, 2012; Dabrowska et al, 2011).

The evaluation of additional senescence markers reads as follows in the result section (page 10) and discussion (page 17):

Results page 10:
To further corroborate that depletion of MKL1/2 induces senescence, we evaluated additional senescence markers for DNA damage and senescence-associated heterochromatic foci (SAHF). We observed p53 Ser15 phosphorylation as an indicator for DNA damage and an accumulation of Histone H3 methylated on lysine 9 in SAHF upon MKL1/2 depletion in HuH7 and HuH6 cells, confirming that these cells are in senescence (Fig. 4F, G).

The induction of senescence requires several secreted factors, referred to as „senescence-messaging secretome (SMS)“ (Kuilman & Peeper, 2009). To identify SMS factors, we performed a microarray in HuH7 and HuH7 MKL1/2 KD cells and found a significant increase in chemokine (C-S-C motif) ligand 10 (CXCL10) and TNFSF10 mRNA expression in HuH7 and HuH6 MKL1/2 KD cells (Fig. 4H and S6).

Discussion page 17:
Further support for the establishment of oncogene-induced senescence upon MKL1/2 depletion comes from the notion of p53 phosphorylation on serine 15 in HuH7 and HuH6 hepatocellular carcinoma cells. Di Micco and colleagues have shown that oncogene-induced senescence is a consequence of the activation of a robust DNA damage response, characterized by phosphorylation of p53 on serine 15 (Di Micco et al, 2006). Furthermore, we observed an accumulation of histone H3 methylated on lysine 9 in HuH7 and HuH6 cells. The replacement of histone H3 acetylation by methylation in senescence-associated heterochromatic foci (SAHF) is another hallmark of senescence (Narita et al, 2003). The induction of senescence requires several secreted factors, collectively termed as "senescence-messaging secretome" (SMS) (Kuilman & Peeper, 2009). Amongst the SMS factors, the expression of the chemokine (C-S-C motif) ligand 10 (CXCL10) and TNFSF10 was significantly increased upon MKL1/2 depletion. Both CXCL10 and TNFSF10 have been shown to drive numerous human cancers into senescence (Braumuller et al, 2013). The evalulation of these additional senescence markers in vitro and in vivo in HuH7 mouse tumor xenografts provides further evidence that depletion of MKL1 and 2 induces senescence.

2. For the in vivo experiments (Figure 6), the number of SAbGal positive cell is of 2% in the ctrl vs 4% siMKL1. This differential is too low to conclude that there is an increased SAbGal activity that might explain the phenotype, in addition this requires other(s) senescence markers.

Response:
The reviewer is right that the difference in Fig. 6D is too low; a clear result is now displayed in revised Fig. 6G.
To further corroborate that depletion of MKL1 and 2 results in senescence induction in vivo, we directly determined $p16^{INK4a}$ expression, the hallmark of senescent cells (Michaloglou et al, 2005). $P16^{INK4a}$ mRNA expression was significantly elevated in tumours of mice treated with MKL1+2 siRNAs (revised Fig. 6D). We were able to verify the other candidate senescence markers from Fig. 4 in vivo, such as phosphorylation of p53 at Ser15 and an accumulation of histone H3 methylated on lysine 9 (H3K9me3) (revised Fig. 6D). CXCL10 mRNA as a component from the senescence-associated secretome, was also strongly enhanced in the MKL1+2-depleted tumours (revised Fig. 6D). Furthermore, we assayed the tumours for the presence of cells that are actively proliferating by measuring Ki-67 levels, and found a strongly reduced Ki-67 mRNA expression in the tumours (revised Fig. 6C). Taken together, these data demonstrate that the regression of the xenografts is associated with senescence.

We have revised Fig. 6 accordingly, inserted new Fig. 6 C (Ki-67 expression) and D (p16, H3K9me3, p53Ser15 and CXCL10 expression) and added this text on page 13 (results) and page 19 (discussion):

Results page 13:
In order to confirm that the regression of the xenografts is associated with senescence in the tumors treated with MKL1+2 siRNA, we determined $p16^{INK4a}$ expression. $P16^{INK4a}$ mRNA expression was significantly elevated in tumors of mice treated with MKL1+2 siRNA. Furthermore, we were able to verify the other candidate senescence markers shown in Fig. 4 in vivo, such as an accumulation of histone H3 methylated on lysine 3 (H3K9me3), phosphorylation of p53 at serine 15 and enhanced CXCL10 expression as a component of the senescence-associated secretome (Fig. 6D).

Discussion page 19:
The regression of the tumor xenografts was associated with senescence, because $p16^{Ink4a}$ mRNA expression was significantly elevated and the candidate senescence markers, such as accumulation of histone H3 methylated on lysine 9, phosphorylation of p53 at serine 15 and enhanced expression of the senescence-associated chemokine CXCL10 mRNA, were detectable in the tumors of the animals treated with MKL1 and 2 siRNA.

Other points:
-Figure 2: nuclear counterstaining is required to conclude about MKL1 localization.
- corrected –

-Figure 4: Ras and its downstream targets activation (Raf, Mek...) are well known inducers of OIS in "normal" primary cells. Its ability to induce OIS in cancer cells is not the rule and is even probably the exception. It will be nice to show that constitutive expression of an active Ras or/and downstream effecters (Raf or Mek) are able to induce OIS in HuH6 and 7 cells.
Efficiency of UO126 treatment should be checked by performing immunoblot against phospho-ERK in MKL1/2 KD cells.
Involvement of p16 should also be check by performing its knockdown in MLK1/2 KD cells.

Response:
To prove experimentally that constitutive expression of active Ras is able to induce OIS in HuH7 cells, we introduced an activated ras allele (H-*ras*V12) into HuH7 cells using retroviral transduction. On the fourth day postinfection, we were able to detect enhanced pERK, p16 expression, hypophosphorylation of Rb and phosphorylation of p53 at Ser15 in HuH7 and HuH6 cells (revised Fig. 4I), indicating that constitutive expression of active Ras induces OIS in HuH6 and HuH7 cells. Due to space restrictions, we left out HuH6 cells and depict selected OIS markers in those cells in Fig. S7.

We commented on these new figures in the text on page 10 (results) and page 21 (materials and methods):

Results page 10:

Since Ras and its downstream targets are well known inducers of oncogene-induced senescence in primary cells, but not in cancer cells, we tested whether expression of active Ras is able to induce oncogene-induced senescence in HuH7 and HuH6 cells by retroviral transduction of an activated ras allele (H-*ras*V12). On the fourth day postinfection, enhanced ERK phosphorylation, p16 expression, hypophosphorylation of Rb and phosphorylation of p53 at Ser15 was detectable in HuH7 and HuH6 cells (Fig. 4I and S7), indicating that constitutive expression of active Ras induces oncogene-induced in HuH6 and HuH7 cells.

Materials and methods page 21:

For the retroviral transduction, amphotropic Phoenix packaging cells were transfected by calcium phosphate DNA precipitation with the pBabe puro H-Ras V12 plasmid. After 48 hours, the virus containing supernatant was filtered (0.45 µM filter; Millipore) and supplemented with 4 µg/mL polybrene (Sigma-Aldrich, Taufkirchen).

-Figure 4: Efficiency of UO126 treatment should be checked by performing immunoblot against phospho-ERK in MKL1/2 KD cells.

Response:

We prepared a corresponding western blot (new Fig. 4C) showing that UO126 treatment prevents phosphorylation of ERK.

-Figure 4: Involvement of p16 should also be check by performing its knockdown in MLK1/2 KD cells.

Response:

To examine whether the expression of $p16^{INK4a}$ is necessary for senescence induction by MKL1/2 depletion, we performed siRNA-mediated p16 knockdown prior to lentiviral transduction with MKL1/2 shRNA and analyzed phosphorylation of Rb. As a result, hypophosphorylation of Rb was observed upon MKL1/2 knockdown, but not upon $p16^{INK4a}$ / MKL1/2 double knockdown. These results suggest that p16 expression is required for senescence induction.

This text can be found on page 10 and the corresponding figure S5.

-Figure 5: to claim that adding DLC1 in DLC1-negative cells induces senescence, the authors should check that the cells stop to proliferate by performing growth curves experiments and display at least one other senescence markers. HuH7DLC1 without dox cells should also be compared to HuHDLC1 cells with dox and not only with HuH7.

Response:

We are grateful to Reviewer 2 for pointing out that the growth curves depending on the DLC1 expression are missing. We added a growth curve in Fig. 5B, displaying DLC1-negative HuH7 with and without a Tet-off regulated promoter to express DLC1. DLC1 expression provokes a proliferation arrest in HuH7 cells, which is released upon switching off DLC1 expression by adding doxycycline. We added the following sentence on page 11: “We observed that HuH7 cells stopped to proliferate upon expression of DLC1”.

copyright EMBO

To further substantiate that the proliferation arrest induced by DLC1 reconstitution is due to senescence induction, we display two additional senescence markers, p53 Ser15 and H3K9me3, in HuH7 and HuH7 DLC1 cells with dox and without doxycycline. In revised Fig. 5D, we compared the remaining senescence markers to HuH7 DLC1 cells with doxycycline.

-Figure 6: tumor volume (6C) include various conditions (siMKL1/2, siMKL1+2, siMKL1) but the experiments around of validation of the tools or analysis were not performed with the 3 conditions. The rationale for this is difficult to understand.
Fig 6C, siMKL1 display a better efficiency to inhibit tumor growth than siMKL1/2 or siMKL1+2, the author should comment and discuss this result and this might mean that for the all in vitro experiments inhibiting MKL1 and 2 was useless and the experiments should have been performed only with MKL1 KD.

Response:

We would like to thank Reviewer 2 for the useful comment. In Fig. 6A, we sought to introduce another treatment group by using MKL1 and MKL2 siRNA-PEI complexes to confirm the strong effect of MKL1/2 on tumor growth. In order to determine to which extent MKL1 siRNA alone contributes to the inhibitory effect of the MKL1 and MKL2 siRNA-PEI complexes, we included a MKL1 siRNA treatment group. Surprisingly, we found that MKL1 siRNA alone was as effective in inhibiting tumor growth as MKL1/2 siRNA. We will be investigating further the relative contribution of MKL1 to hepatocarcinogenesis, which is beyond the scope of this paper.

Referee #3: Major Concerns:

1. There is a lack of experimental detail, particularly with regard to the 'n' for the experiments shown. This should be clarified throughout and all experiments showing statistical significance need to have been performed a minimum of 2 times.

Response:

We apologize for the lack of experimental detail. The figure legends of the revised paper contain now information about the number of experiments and the statistical analysis.

2. In Figure 1, the investigators should show a western blot of the cell lines expressing roughly equivalent amounts of MKL1/2. Also is Fi 1, better labels are needed on figures (e.g. For Fig 1B, under controls maybe put Scr; for Fig 1E, mark as DLC1-deficient cell lines, and for 1F, indicate that these are DLC1+ cell lines.

Response:

We have added a western blot displaying the MKL1/2-expression in HuH7, HuH6, HLF and HepG2 cells in revised Fig. 1A and inserted the abbreviation ctrl under the control cells. For reasons of clarity, we labeled the HuH7 and HuH6 cells as DLC1-deficient and the HLF and HepG2 cells as DLC1-expressing cells.

3. For MKL1 mRNA expression, what is it relative to (what exactly is the y-axis)? The control in the SM22 graph is 1, which makes sense, but the controls in the MKL1 graphs are not 1.

Response:

MKL1 mRNA expression was determined by quantitative real time RT-PCR using 18S rRNA levels for normalization. Data are mean s.d. from triplicate experiments. Since each of the three

experiments was performed in duplicates and one value of the control set to 1, the control scatters around 1 (HuH7 ctrl: 0.98 ± 0.16; HLF ctrl: 0.84 ± 0.22; HepG2 ctrl: 1.06 ± 0.08).

4. If HepG2 and HLF do not have nuclear MKL1, then they have no active MKL1 to inhibit. This should be clarifies for the readers.

We have clarified this issue for the readers by adding one sentence on page 6 that reads:
"Therefore HLF and HepG2 cells have little active nuclear MKL1/2".

5. For the data shown in Figure 2, why did you use a Tet-off system if you are not taking advantage of this? (e.g. performing experiments on identical calls + and - dox and show that MKL1 returns to cytoplasm? DAPI staining should be shown for Fig 2B. For Fig 2D, the investigators should show controls without active RhoA. Is the active RhoA affecting cell growth compared to controls? Also, is ther partial rescue of HuH6/7 + MKL1/2 KD + RhoAV14?

Response:
In the revised manuscript, we took advantage of the Tet-off system and show that MKL1 returns to the cytoplasm upon treatment with doxycycline in revised Fig. 2C and modified the text on page 7 accordingly. We also inserted a DAPI staining in Fig. 2 and a control graph without active RhoA in Fig. 2D. There was a significant increase in cell proliferation in RhoAV14-expressing and control HepG2 cells. As suggested by the reviewer, we also introduced RhoAV14 into HuH7 cells and found no difference in cell proliferation between HuH7 and HuH7 RhoAV14-expressing cells. This is likely to be due to the constitutive activation of RhoA in HuH7 cells.

6. For the data shown in Figure 4, HepG2 should also be quantified in Fig 4A. Also, the authors should label IP and IB. Also, the authors should indicate and discuss that U0126 can also affect mTOR.

Response:
We quantified HepG2 in Fig. 4A and labeled the IP and IB. To indicate that UO126 can also affect mTOR, we added the following sentence on page 15:

According to a previous study, UO126 suppresses senescence by inhibiting the MEK/ mTOR pathway (Demidenko et al, 2009). MTOR might therefore also contribute to the pro-proliferative effect of U0126 in HuH7 MKL1/2 KD cells.

7. For the data shown in Figure 5, does re-expression of DLC1 overcome MKL1/2 KD and prevent the anti-proliferative effect of KD?

Response:
Based on our findings, both DLC1 expression and MKL1/2 KD have an antiproliferative effect. We therefore didn't perform this experiment.

8. For the data shown in Figure 6, an excellent control would be HepG2 xenografts not responding to the MKL1/2 KD.

copyright EMBO

Response:

We agree with Reviewer 3 that HepG2 xenografts not responding to the MKL1/2 KD would be a nice control. In an attempt to address this issue, we performed soft agar assays with HepG2 and HepG2 MKL1/2 KD cells and found that depletion of MKL1/2 didn't affect HepG2 colony formation on soft agar. Since the proliferative potential of HepG2 and HepG2 MKL1/2 KD cells also remained unchanged, we decided not to perform HepG2 xenografts for ethical reasons. The results from the soft agar assay and the proliferation assay strongly suggest, however, that HepG2 xenografts would not respond to the MKL1/2 KD.

Referee #3: Minor Concerns:

1. Why were some standard techniques not used - GTPase pull down, Ki67?

Response:

When we assessed the RhoA activation status in our last paper, we used different techniques (Muehlich et al, 2012). Out of these, immunoprecipitation with an antibody that specifically recognizes GTP-bound RhoA proved to be most reliable. Therefore we chose the same approach for determining the Ras activation status in the present paper. We thank Reviewer 3 for suggesting to analyze Ki-67 expression. We found a significant lower Ki-67 expression in the tumors treated with MKL1/2 siRNA as opposed to the tumors treated with control siRNA (see revised Fig. 6C).

2. What is the prognostic significance of DLC1+ versus deficient HCC - can prognosis be subclassified based on DLC1 or MKL1/2 expression/dependence?

Response:

We agree with the reviewer that prognostic relevance of DLC1 and MKL1/2 is of interest. Unfortunately, for the HCC cohort where the expression of both factors was previously analyzed (Muehlich et al, 2012) no survival data is/was available. In addition, meaningful survival data for HCC requires careful selection of the patients because besides the tumor itself (size, number of tumors, etc) the stage of the underlying liver disease and thereby the function of the remaining liver after partial hepatectomy is an important prognostic factor and should be similar among the patients analyzed. Moreover, explanted livers of HCC patients who underwent liver transplantation is a valuable source for HCC tissue and represents a significant fraction of HCCs in the cohort mentioned above. However, overall survival of these patients is primarily affected by the quality of the donor liver, post-transplantation management, etc and is therefore less informative in the given context.

3. What about the MKL1 inhibitor CCG-1423? Can this be used in early stage cirrhotic lesions to prevent MKL1/2 nuclear localization, and therefore prevent progression to HCC?

Response:

We thank the reviewer for this excellent suggestion. CCG-1423 was able to prevent MKL1/2 nuclear localization in HuH7 cells (Fig. R7, this rebuttal), suggesting that it might indeed be a useful therapeutic strategy to prevent progression to HCC. Future studies will need to explore CCG-1423 treatment in patients with cirrhotic lesions.

4. Knockdown of MKL1 systemically as a therapeutic strategy might have systemic effects. Minimally, the investigators should indicate what toxicities they assessed for, and the discussion should include potential side effects of systemically blocking MLK1 and 2 (e.g. platelet counts, hepatocyte maturation)

Response:

We assessed for liver toxicity by histological evaluation of the livers of mice treated with MKL1/2 siRNA (revised Fig. S10). Importantly, no signs of liver damage such as imflammation, necrosis, steatosis or fibrosis were detected. This is stated on page 19 and page 13 (last sentence).

5. How might these results link with the role of MKL1 in leukemia?

Response:

MKL1 was originally identified at a t(1;22) chromosomal translocation of megakaryoblastic leukemia (Ma et al, 2001; Mercher et al, 2001). MKL1 was fused with the RBM15 gene (a.k.a. OTT) to form an RBM15-MKL1 fusion protein, which localizes to the nucleus and constitutively activates SRF and its target genes (Descot et al, 2008). This is reminiscent of the situation in HuH7 cells, where MKL1/2 are constitutively localized to the nucleus and activate MKL/SRF target genes (Muehlich et al, 2012). Given that the leukemia has been shown to be caused by activation of SRF target genes, the potential for a MKL1/2 targeted therapy hold prospects.

We inserted the following sentence in the introduction (page 4):

This is reminiscent of the MKL1 fusion protein with the RBM15 gene (a.k.a. OTT), which is a translocation found in acute megakaryoblastic leukemia. RBM15-MKL1 is constitutively nuclear and activates SRF and its target genes (Descot et al, 2008; Ma et al, 2001; Mercher et al, 2009).

6. Does DLC1 deficiency affect Ras activity? Is Ras mutated in any of these cell lines employed?

Response:

We found that DLC1 deficiency didn't affect Ras activity. Out of the four cell lines, one (HepG2) has a N-ras mutation at codon 61 position 2 (Hsu et al, 1993).

7. For completeness, Fig 1 should include MKL1 mRNA for the HuH6 cell line.

- corrected-

8. In Figure 3, the authors need to clarify what day after MKL1/2 KD the studies were performed.

Response:

The studies in Fig. 3 were performed at the 7th day postinfection. This is now indicated in the Figure legends.

9. In figure 6D, why were other siRNA conbinations not shown and quantitated? How were the 5 40x fields per sample collected? What is the n? Why is there no error bar for the third column

Response:

We were able to obtain tumor material only in mice treated with MKL1+2 siRNA. There was a complete regression of xenografts in the MKL1/2 and MKL1 siRNA treatment groups. The 5 40x fields were randomly collected. There is no error bar for the third column, because only one out of 6 animals treated with MKL1+2 siRNA had a tumor.

R1

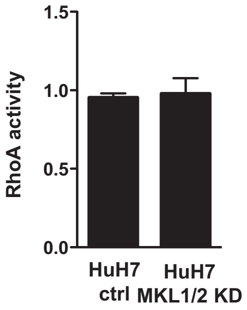

Figure R1. Active RhoA-GTP levels of HuH7 cells expressing control shRNA or MKL1/2 shRNA were assessed by an ELISA assay. Values are mean ± SD of three independent experiments.

R2

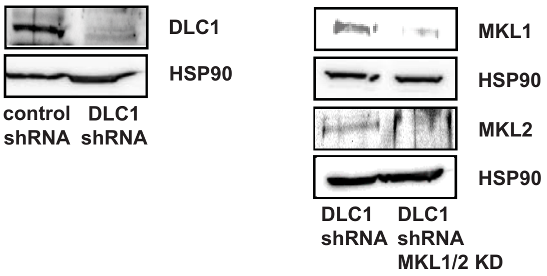

Figure R2. Hepatocytes stably expressing control shRNA or DLC1 shRNA were subjected to immunoblotting using anti-DLC1 and anti-HSP90 antibodies. Hepatocytes expressing DLC1 shRNA and MKL1/2 shRNA were immunoblotted with anti-MKL1, anti-MKL2 and anti-HSP90 antibodies.

R3

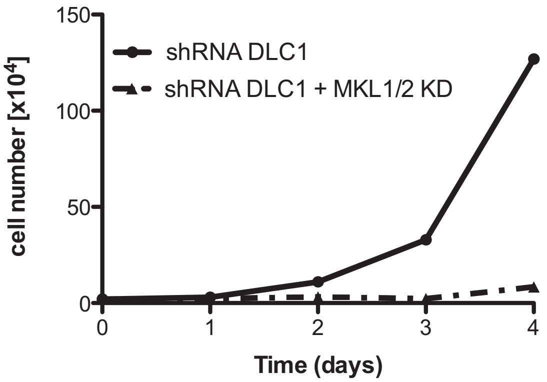

Figure R3. Hepatocytes stably expressing DLC1 shRNA were transduced with control shRNA and MKL1/2 shRNA. Cells were counted daily for 4 days.

copyright EMBO

R4

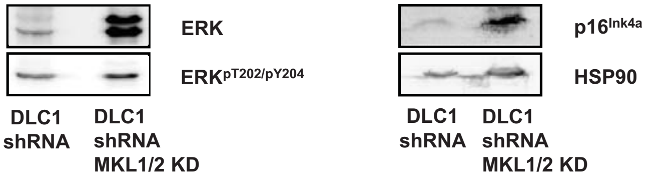

Figure R4. Lysates of the indicated cell lines were immunoblotted with anti-ERK$^{pT202/pY204}$, total anti-ERK, anti-p16^{Ink4a} and anti-HSP90 antibodies.

R5

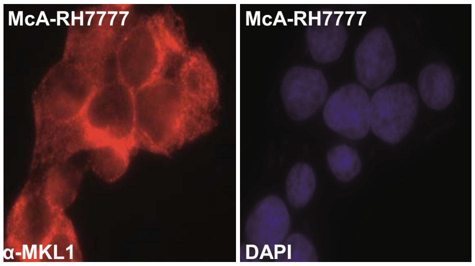

Figure R5. McA-RH7777 cells were stained with anti-MKL1 antibody and nuclei were counterstained with DAPI for immunoflourescence analysis.

R6

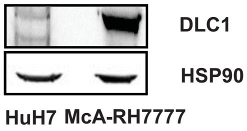

Figure R6. Lysates of HuH7 and McA-RH7777 cells were subjected to immunoblotting using anti-DLC1 and anti-HSP90 antibodies.

R7

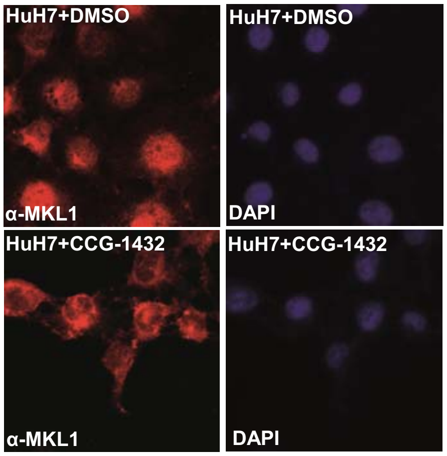

Figure R7. HuH7 cells were treated with 10 μM CCG-1432 for 24 hours and immunostained with anti-MKL1 antibody. Nuclei were counterstained with DAPI.

References:

Braumuller H, Wieder T, Brenner E, Assmann S, Hahn M, Alkhaled M, Schilbach K, Essmann F, Kneilling M, Griessinger C, Ranta F, Ullrich S, Mocikat R, Braungart K, Mehra T, Fehrenbacher B, Berdel J, Niessner H, Meier F, van den Broek M, Haring HU, Handgretinger R, Quintanilla-Martinez L, Fend F, Pesic M, Bauer J, Zender L, Schaller M, Schulze-Osthoff K, Rocken M (2013) T-helper-1-cell cytokines drive cancer into senescence. *Nature* **494**(7437)**:** 361-365

Cahu J, Bustany S, Sola B (2012) Senescence-associated secretory phenotype favors the emergence of cancer stem-like cells. *Cell Death Dis* **3:** e446

Cen B, Selvaraj A, Burgess RC, Hitzler JK, Ma Z, Morris SW, Prywes R (2003) Megakaryoblastic leukemia 1, a potent transcriptional coactivator for serum response factor (SRF), is required for serum induction of SRF target genes. *Mol Cell Biol* **23**(18)**:** 6597-6608

Dabrowska M, Skoneczny M, Rode W (2011) Functional gene expression profile underlying methotrexate-induced senescence in human colon cancer cells. *Tumour Biol* **32**(5)**:** 965-976

Demidenko ZN, Shtutman M, Blagosklonny MV (2009) Pharmacologic inhibition of MEK and PI-3K converges on the mTOR/S6 pathway to decelerate cellular senescence. *Cell Cycle* **8**(12)**:** 1896-1900

Descot A, Hoffmann R, Shaposhnikov D, Reschke M, Ullrich A, Posern G (2009) Negative regulation of the EGFR-MAPK cascade by actin-MAL-mediated Mig6/Errfi-1 induction. *Mol Cell* **35**(3)**:** 291-304

Descot A, Rex-Haffner M, Courtois G, Bluteau D, Menssen A, Mercher T, Bernard OA, Treisman R, Posern G (2008) OTT-MAL is a deregulated activator of serum response factor-dependent gene expression. *Mol Cell Biol* **28**(20)**:** 6171-6181

Di Micco R, Fumagalli M, Cicalese A, Piccinin S, Gasparini P, Luise C, Schurra C, Garre M, Nuciforo PG, Bensimon A, Maestro R, Pelicci PG, d'Adda di Fagagna F (2006) Oncogene-induced senescence is a DNA damage response triggered by DNA hyper-replication. *Nature* **444**(7119)**:** 638-642

Hsu IC, Tokiwa T, Bennett W, Metcalf RA, Welsh JA, Sun T, Harris CC (1993) p53 gene mutation and integrated hepatitis B viral DNA sequences in human liver cancer cell lines. *Carcinogenesis* **14**(5)**:** 987-992

Kuilman T, Peeper DS (2009) Senescence-messaging secretome: SMS-ing cellular stress. *Nat Rev Cancer* **9**(2)**:** 81-94

Lahoz A, Hall A (2008) DLC1: a significant GAP in the cancer genome. *Genes Dev* **22**(13)**:** 1724-1730

Ma Z, Morris SW, Valentine V, Li M, Herbrick JA, Cui X, Bouman D, Li Y, Mehta PK, Nizetic D, Kaneko Y, Chan GC, Chan LC, Squire J, Scherer SW, Hitzler JK (2001) Fusion of two novel genes, RBM15 and MKL1, in the t(1;22)(p13;q13) of acute megakaryoblastic leukemia. *Nat Genet* **28**(3)**:** 220-221

Mercher T, Coniat MB, Monni R, Mauchauffe M, Nguyen Khac F, Gressin L, Mugneret F, Leblanc T, Dastugue N, Berger R, Bernard OA (2001) Involvement of a human gene related to the Drosophila spen gene in the recurrent t(1;22) translocation of acute megakaryocytic leukemia. *Proc Natl Acad Sci U S A* **98**(10)**:** 5776-5779

Mercher T, Raffel GD, Moore SA, Cornejo MG, Baudry-Bluteau D, Cagnard N, Jesneck JL, Pikman Y, Cullen D, Williams IR, Akashi K, Shigematsu H, Bourquin JP, Giovannini M, Vainchenker W, Levine RL, Lee BH, Bernard OA, Gilliland DG (2009) The OTT-MAL fusion oncogene activates RBPJ-mediated transcription and induces acute megakaryoblastic leukemia in a knockin mouse model. *J Clin Invest* **119**(4)**:** 852-864

copyright EMBO

Michaloglou C, Vredeveld LC, Soengas MS, Denoyelle C, Kuilman T, van der Horst CM, Majoor DM, Shay JW, Mooi WJ, Peeper DS (2005) BRAFE600-associated senescence-like cell cycle arrest of human naevi. *Nature* **436**(7051)**:** 720-724

Morin P, Flors C, Olson MF (2009) Constitutively active RhoA inhibits proliferation by retarding G(1) to S phase cell cycle progression and impairing cytokinesis. *Eur J Cell Biol* **88**(9)**:** 495-507

Muehlich S, Hampl V, Khalid S, Singer S, Frank N, Breuhahn K, Gudermann T, Prywes R (2012) The transcriptional coactivators megakaryoblastic leukemia 1/2 mediate the effects of loss of the tumor suppressor deleted in liver cancer 1. *Oncogene* **31**(35)**:** 3913-3923

Narita M, Nunez S, Heard E, Lin AW, Hearn SA, Spector DL, Hannon GJ, Lowe SW (2003) Rb-mediated heterochromatin formation and silencing of E2F target genes during cellular senescence. *Cell* **113**(6)**:** 703-716

Shinozaki K, Ebert O, Kournioti C, Tai YS, Woo SL (2004) Oncolysis of multifocal hepatocellular carcinoma in the rat liver by hepatic artery infusion of vesicular stomatitis virus. *Mol Ther* **9**(3)**:** 368-376

Xue W, Krasnitz A, Lucito R, Sordella R, Vanaelst L, Cordon-Cardo C, Singer S, Kuehnel F, Wigler M, Powers S, Zender L, Lowe SW (2008) DLC1 is a chromosome 8p tumor suppressor whose loss promotes hepatocellular carcinoma. *Genes Dev* **22**(11)**:** 1439-1444

2nd Editorial Decision 11 June 2013

Thank you for the submission of your revised manuscript to EMBO Molecular Medicine. We have now received the enclosed reports from the Reviewers that were asked to re-assess it. As you will see the reviewers are now globally supportive and I am pleased to inform you that we will be able to accept your manuscript pending the following final amendments:

Reviewer 3 feels that the manuscript would be enhanced by a model indicating the proposed connections that are not yet understood - for example, how loss of MKL1 and 2 leads to Rho activation. I think this is a good idea that would improve the impact of your work and I therefore encourage you to do this. I will make an Editorial decision on your revised manuscript and will thus not be sending out the manuscript to the Reviewer again.

Please submit your revised manuscript within two weeks. I look forward to seeing the revised, final form of your manuscript as soon as possible.

***** Reviewer's comments *****

Referee #1 (Remarks):

The revision has greatly improved the manuscript and I recommend its publication in the present form.

Referee #2 (Remarks):

the authors have adressed the various points I have raised during the review process.

Referee #3 (Remarks):

In this revision, the authors have addressed most of my concerns. There are still many unanswered questions/unknown links in their data.

copyright EMBO

I would ask for one more modification - the paper would be greatly enhanced by a model of what they think is going on with question marks indicating the proposed connections that are not yet understood - for example, how loss of MKL1 and 2 leads to Rho activation.

The strength of the paper is this that they have shown using cell lines that when a cell line is dependent on loss of LC1 for proliferation, then this proliferation requires MKL1 and 2. Unfortunately, they showed already (Oncogene 2012). So, this additional information is not great. What is new in this manuscript is that the authors determined that the growth inhibition is not via apoptosis, but via cell cycle arrest and senescence. In this revision, the senescence phenotype was further proven.

2nd Revision - authors' response 17 June 2013

Thank you for the decision letter. I'm glad that you will be able to accept our manuscript. Please find enclosed a model indicating the proposed connections between MKL1/2 loss and senescence induction. It is inserted as new Fig. 7 in the revised manuscript that I submitted yesterday. The corresponding figure legend (see attachment) on the last page of the revised manuscript reads as follows:
Figure 7

Proposed model for senescence induction upon MKL1/2 depletion: DLC1 loss results in RhoA activation and MKL1/2 nuclear localization. Depletion of MKL1/2 induces cellular senescence via activation of the Ras/MAPK and p16INK4a/pRb pathways. It is currently unclear how MKL1/2 depletion causes Ras activation, however, impaired expression of MKL target genes such as mig6, a negative regulator of the EGFR-MAPK cascade, could mediate Ras activation.

Please let me know if Fig. 7 needs another modification. I look forward to hearing from you.

3rd Editorial Decision 20 June 2013

Thank you for re-submitting your manuscript to EMBO Molecular Medicine. Please refer to the above altered number in any correspondence regarding this manuscript.

However, checking your manuscript files I noticed that the resolution of the gels in your supplementary information is really low. Please try to improve their quality and send us a new supplementary information PDF via email to contact@embomolmed.org.

Thank you very much in advance.

We will now re-evaluate your submission and will contact you again on completion.

Additional correspondence - author 21 June 2013

Thank you for your email. We improved the quality of the resolution of the gels in our supplementary information. Please find the revised supplementary information attached to this email.
