# Supplementary material for: Depletion of the transcriptional coactivators megakaryoblastic leukaemia 1 and 2 abolishes hepatocellular carcinoma xenograft growth by inducing oncogene-induced senescence
Source: EMBO Mol Med. 2013 Jul 29;5(9):1367–82. doi: 10.1002/emmm.201202406 (PMC3799492; doi:10.1002/emmm.201202406)
Supplement: Supplementary file 2 [file emmm0005-1367-SD2.pdf]

Supporting Information

Table of Content

1) **Supporting Information Figure 1……………………………… page 2**

2) **Supporting Information Figure 2……………………………….page 2**

3) **Supporting Information Figure 3……………………………….page 3**

4) **Supporting Information Figure 4……………………………….page 3**

5) **Supporting Information Figure 5……………………………….page 4**

6) **Supporting Information Figure 6……………………………….page 4**

7) **Supporting Information Figure 7……………………………….page 4**

8) **Supporting Information Figure 8……………………………….page 5**

9) **Supporting Information Figure 9……………………………….page 5**

10) **Supporting Information Figure 10…………………………...page 6**

S1

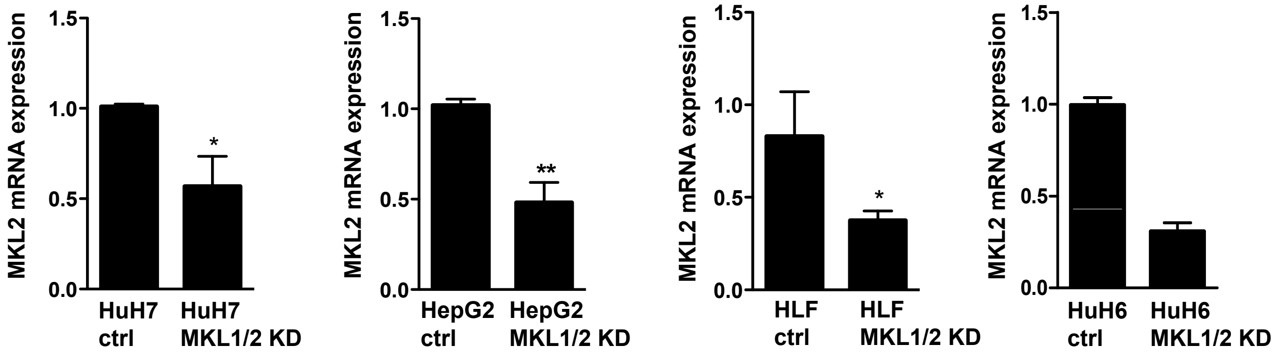

Supporting Information Figure 1. Total RNA was isolated from HuH7, HepG2, HLF and HuH6 cells expressing control shRNA or MKL1/2 shRNA and mRNA levels were measured by qRT-PCR. Values are mean ± SD of three independent experiments or two independent experiments for the right graph; **p = 0.04; **p = 0.0013; *p = 0.015.*

S2

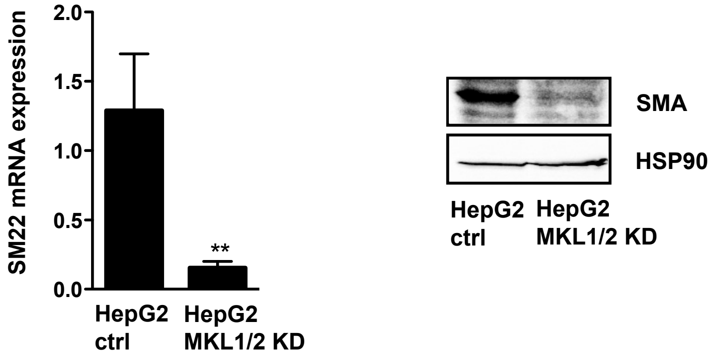

Supporting Information Figure 2. Lysates of HepG2 and HepG2 MKL1/2 KD cells were immunoblotted with anti-SMA and anti-HSP90 antibodies. Relative SM22 mRNA expression was determined by qRT-PCR. Values are mean ± SD (n=3); ***p = 0.003.*

S3

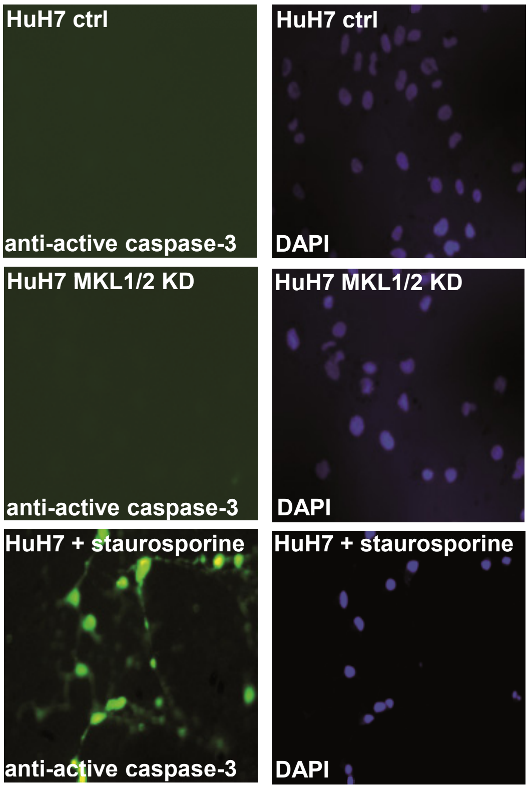

Supporting Information Figure 3. HuH7 and HuH7 MKL1/2 KD cells were treated with and without 1μM staurosporine for 16 hours and subjected to immunofluorescence analysis with anti-active caspase-3 antibody and DAPI for nuclear counter staining.

S4

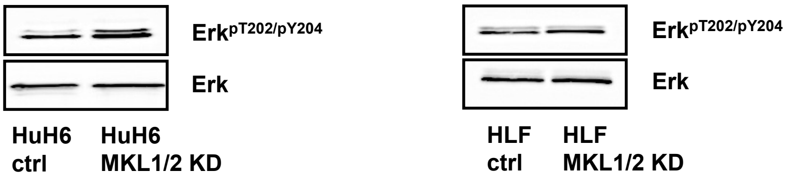

Supporting Information Figure 4. HuH6, HuH6 MKL1/2 KD, HLF and HLF MKL1/2 KD cells were immunoblotted with anti-ERK$^{pT202/pY204}$ and total anti-ERK antibodies, respectively.

S5

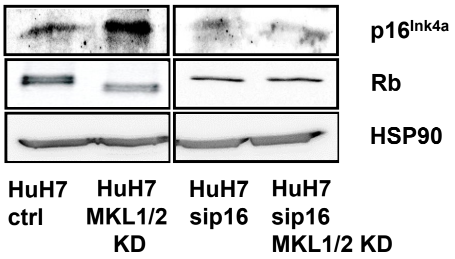

Supporting Information Figure 5. HuH7 cells were transfected with p16^{Ink4a} siRNA (50 nM) and after 24 hours transduced with control shRNA or MKL1/2 shRNA. At day 5 postinfection, cells were lysed and subjected to immunoblotting using anti-p16^{Ink4a}, anti-Rb and anti-HSP90 antibodies.

S6

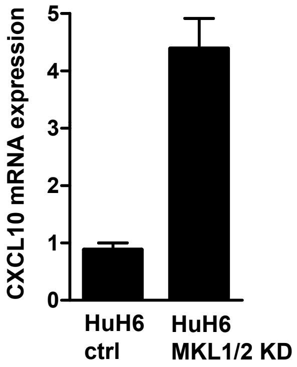

Supporting Information Figure 6. Relative CXCL10 mRNA expression of HuH6 cells expressing control shRNA or MKL1/2 shRNA was determined by qRT-PCR. Values are mean ± SD (n=2).

S7

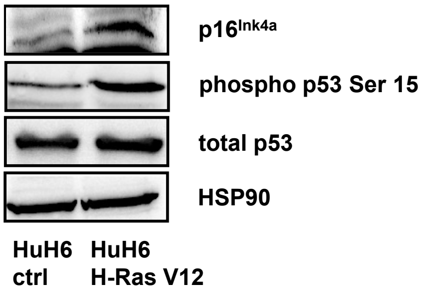

Supporting Information Figure 7. HuH6 cells were infected with pBabe H-Ras V12 or pBabe control vector and at day 4 postinfection cells were immunoblotted with anti-p16^{Ink4a}, anti-p53 phospho serine 15, total anti-p53 and anti-HSP90 antibodies.

S8

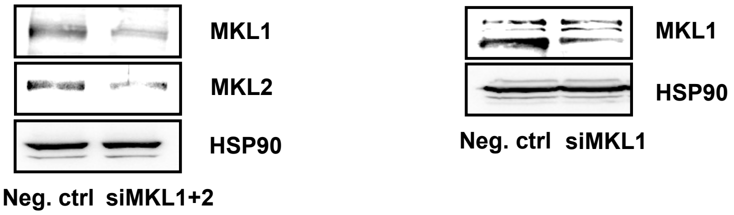

Supporting Information Figure 8. HuH7 cells were transfected with either Neg. ctrl siRNA, MKL1 siRNA or a combination of MKL1 and MKL2 siRNAs. After 48 hours, lysates were immunoblotted with anti-MKL1, anti-MKL2 and anti-HSP90 antibodies.

S9

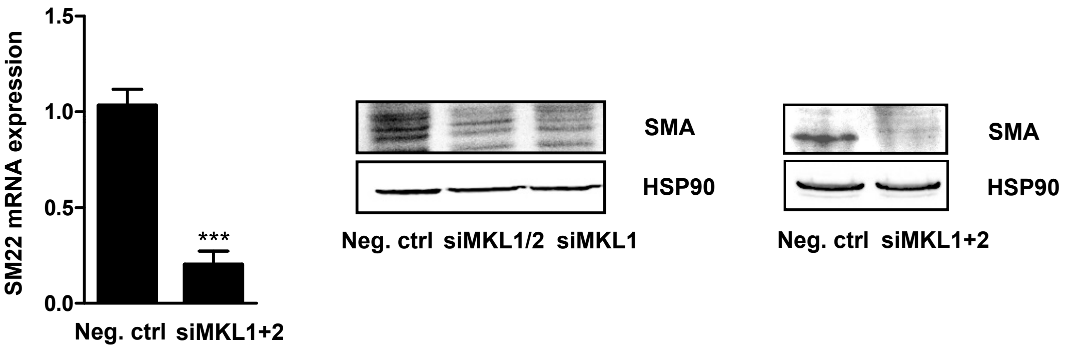

Supporting Information Figure 9. SM22 mRNA expression of HuH7 cells transfected with Neg. ctrl siRNA or MKL1+2 siRNA was determined by qRT-PCR. Values are mean ± SD (n=3); ****$p < 0.0001$.* Lysates of HuH7 cells expressing Neg. ctrl siRNA, MKL1/2 siRNA, MKL1 siRNA or a combination of MKL1 and MKL2 siRNAs were immunoblotted with anti-SMA and anti-HSP90 antibodies.

S10

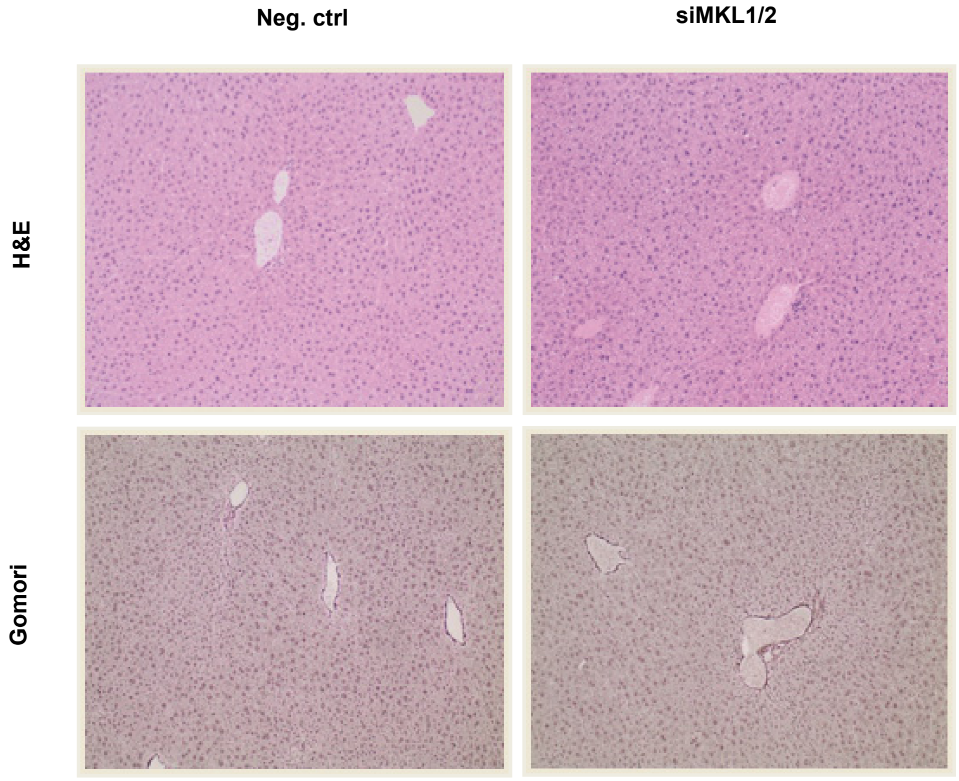

Supporting Information Figure 10. H&E and Gomori stains of liver tissue from mice treated with Neg. ctrl or MKL1/2 siRNA. Both conditions show regular murine liver parenchyma.
